# Improving the Precision of Base Editing by Bubble Hairpin Single Guide RNA

**DOI:** 10.1128/mBio.00342-21

**Published:** 2021-04-20

**Authors:** Zhiwei Hu, Yannan Wang, Qian Liu, Yan Qiu, Zhiyu Zhong, Kangdi Li, Wenhua Li, Zixin Deng, Yuhui Sun

**Affiliations:** a Key Laboratory of Combinatorial Biosynthesis and Drug Discovery (Ministry of Education), School of Pharmaceutical Sciences, Wuhan University, Wuhan, People’s Republic of China; b Hubei Key Laboratory of Cell Homeostasis, College of Life Sciences, Wuhan University, Wuhan, People’s Republic of China; NIAID, NIH

**Keywords:** CRISPR base editor, adenosine deaminase, bubble hairpin sgRNA, cytidine deaminase

## Abstract

Base editing is a powerful genome editing approach that enables single-nucleotide changes without double-stranded DNA breaks (DSBs). However, off-target effects as well as other undesired editings at on-target sites remain obstacles for its application. Here, we report that bubble hairpin single guide RNAs (BH-sgRNAs), which contain a hairpin structure with a bubble region on the 5′ end of the guide sequence, can be efficiently applied to both cytosine base editor (CBE) and adenine base editor (ABE) and significantly decrease off-target editing without sacrificing on-target editing efficiency. Meanwhile, such a design also improves the purity of C-to-T conversions induced by base editor 3 (BE3) at on-target sites. Our results present a distinctive and effective strategy to improve the specificity of base editing.

## Abstract (precis)

Base editors are double-strand-break (DSB)-free genome editing tools and have been widely used in diverse living systems. However, it is reported that these tools can cause substantial off-target editing.

## INTRODUCTION

Base editors (BEs) are CRISPR RNA-guided programmable deaminases which are able to efficiently convert base pair C:G to T:A (CBE) or base pair A:T to G:C (ABE) without inducing double-strand breaks (DSBs) or providing donor DNA templates (1, 2). Base editing systems are composed of three parts: the catalytically inactive nuclease Cas9 (dCas9 or nCas9), the deaminase, and the single guide RNA (sgRNA) (1, 2). Owing to their high base conversion efficiency and low indel rate, base editors have been widely used in various living systems including cultured cells (1–7) and whole organisms (8, 9). However, it has been reported that BEs can generate substantial undesired editings on DNA and RNA (10–18). To improve the utility of BEs, several strategies have been used, including Cas protein evolution (7, 14), deaminase protein engineering (2, 19, 20), and sgRNA modification (11, 13), and there is still huge potential for improvement.

**FIG 1:**
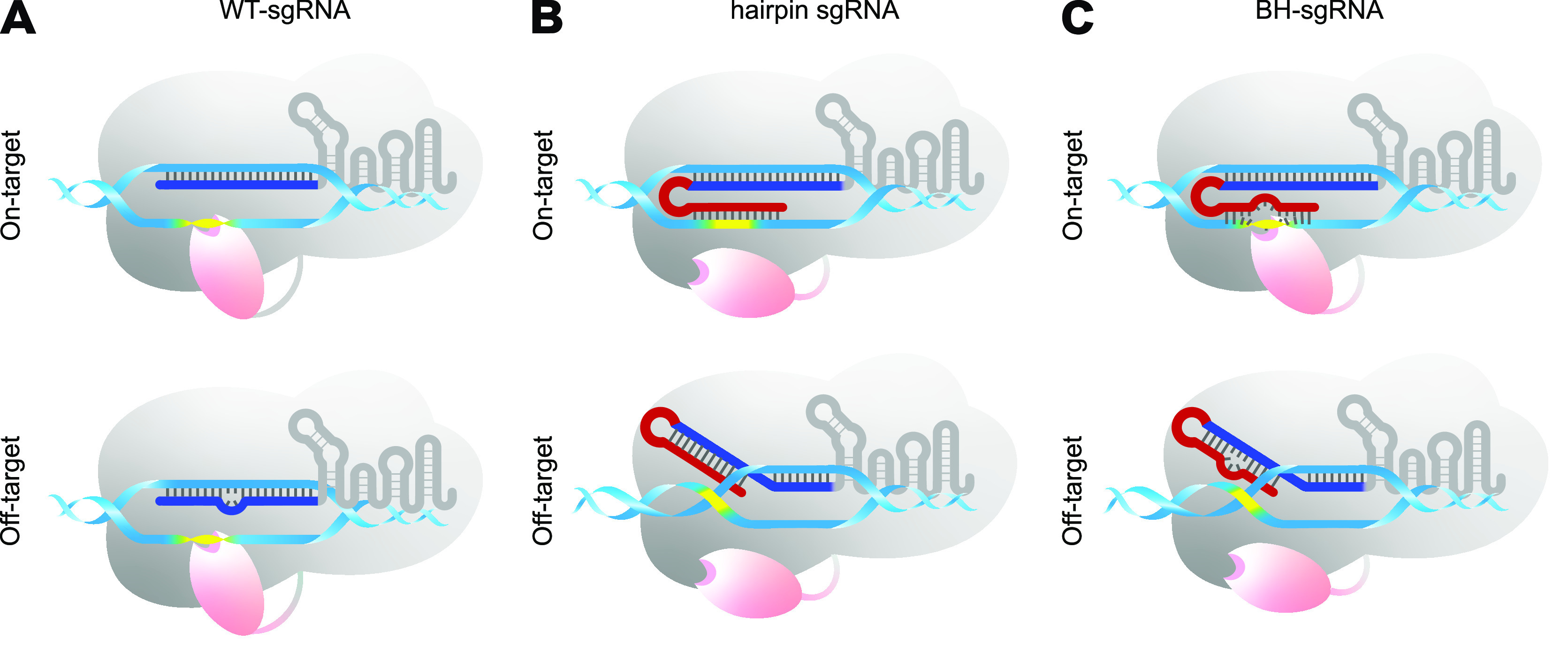
Scheme of BE with different sgRNAs. Schematic representation of BE with WT-sgRNA (A), hairpin sgRNA (B), and BH-sgRNA (C) at on- and off-target sites. Spacer sequence and the hairpin sequence are highlighted in blue and red, respectively. The deaminase and Cas9 are shadowed in pink and gray, respectively. The editing window is shown in yellow.

Previous reports have demonstrated that sgRNA length could affect the editing windows and the specificity of BEs (21). Extended sgRNAs containing one or two extra guanines at the 5′ terminus improved the specificity of BEs to a certain extent, while truncated sgRNAs showed an unclear effect (11, 13). The recent reported hairpin secondary structures of sgRNA can increase cleavage specificity of Cas9 by influencing the R-loop complete formation (22). It inspired us to introduce the secondary structure of sgRNA to BEs. It is known that the complete formation of the RNA-DNA R-loop is the most vital step in the base editing process, for only in this way can the single-stranded DNA substrate be fully exposed to deaminase (Fig. 1A) (1, 2). We reasoned that the accessibility of deaminase could be regulated by sgRNA secondary structures to improve BE specificity. However, the function of sgRNA in BEs is as not only a guide but also a support for single-stranded DNA exposure, which is different from other Cas9-directed tools. Based on this function difference, we assumed that if we directly adapted hairpin sgRNAs to BEs, the perfect complementarity between extended sequence and target DNA would promote DNA-RNA duplex formation (23), which might prevent nucleotides within the editing window from being fully exposed to deaminase even though the R-loop would be successfully formed (Fig. 1B). Thus, we designed a distinctive bubble hairpin sgRNA (BH-sgRNA) containing a 5′ extended sequence complementary to the guide sequence and several mismatches in the extended sequence which would pair with the editing window (Fig. 1C). We supposed that the heteroduplex formed by pairing between the added complementary sequence and the guide sequence could provide an energetic and steric barrier for complete R-loop formation at off-target sites. In contrast, at on-target sites the target DNA perfectly matches the guide sequence, allowing hairpin disruption and complete R-loop formation (Fig. 1).

## RESULTS

### Structure design and optimization of sgRNA.

**FIG 2:**
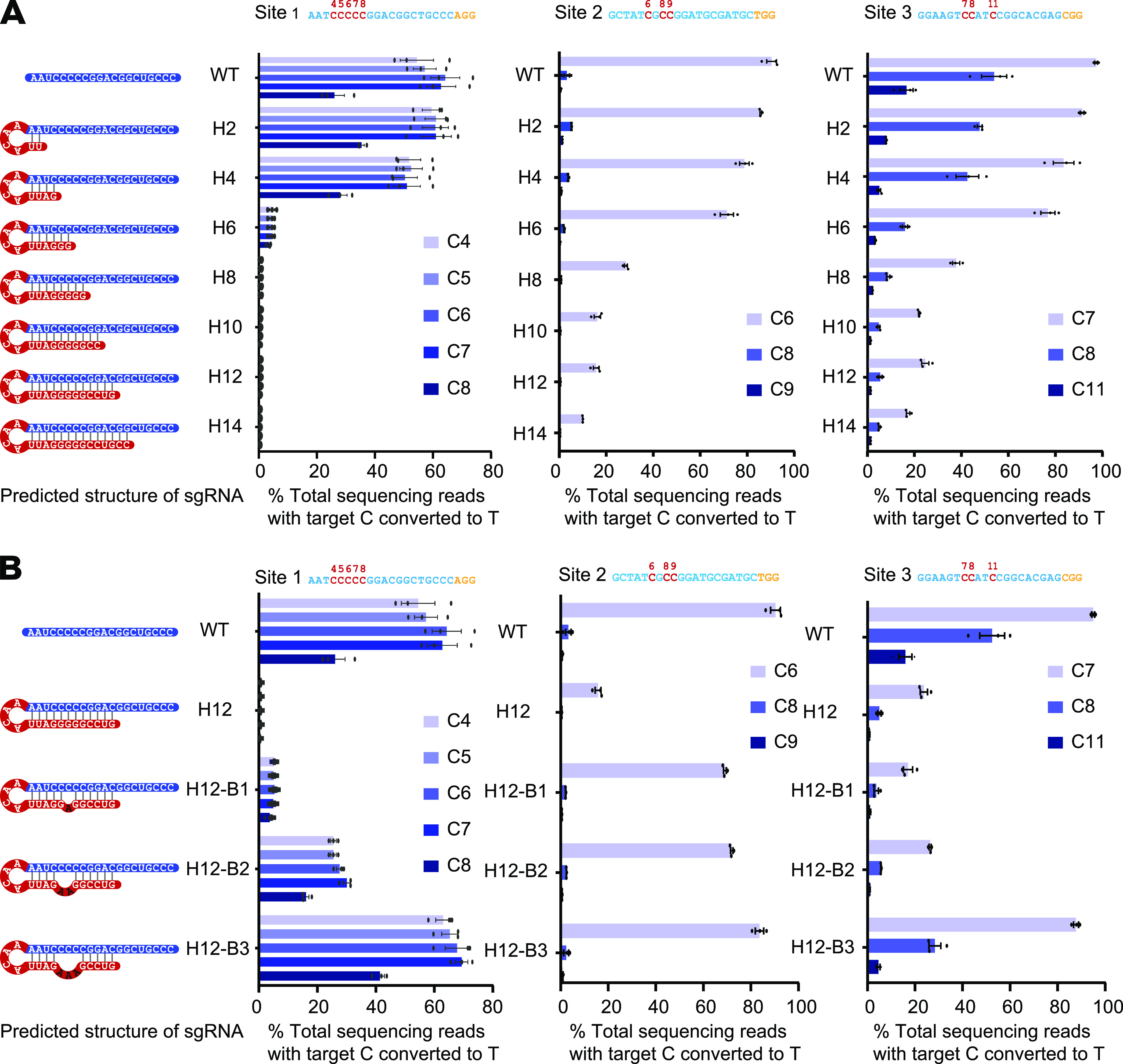
Activity of hairpin BE3 with sgRNA and BH-sgRNA. (A) Base editing efficiency using hairpin sgRNAs with different hairpin lengths. Predicted structures of hairpin sgRNAs are shown on the left, followed by three bar charts showing on-target editing efficiency at sites 1, 2, and 3, respectively. The labels along the vertical axis correspond to predicted structures of sgRNA; for example, H2 represents hairpin sgRNA with 2 nt added on the 5′ end of the spacer excluding the 4-nt loop. (B) Base editing efficiency using BH-sgRNAs; bubble size varied from 1 to 3 nt. Predicted structures of BH-sgRNAs are shown on the left, followed by three bar charts showing on-target editing efficiency at sites 1, 2, and 3. H12-B3 represents BH-sgRNA with a 12-nt hairpin and 3-nt bubble. “C[number]” refers to the “C” position in the target sequence (counting the end distal to the PAM as position 1). Values and error bars reflect mean ± SEM for three independent biological replicates performed on different days. Individual means and *P* values are listed in Table S1.

In our RNA hairpin design, a 5′-ACAA-3′ tetraloop was introduced into hairpin sgRNA. As such a structure has been found to introduce a sharp turn into a natural RNA helix (22, 24), we expect that it could promote pairing between extended complementary sequence and the sgRNA guide sequence. We constructed a series of hairpin sgRNAs with different hairpin lengths and measured the activities of these sgRNAs by detecting BE3-induced nucleotide change at three target sites in Escherichia coli. Deep sequencing results suggested there was no obvious difference in base editing efficiency when complementary sequence length was no more than 4 nucleotides (nt), but the editing efficiency decreased dramatically with further increase of hairpin length (Fig. 2A; see also Fig. S1 in the supplemental material). This contrasts with Cas9, where the editing efficiency is still considerable even when the complementary sequence length reaches 12 nt (22, 25). This result complied with our prediction that the hairpin would block deaminase activity through interfering with complete R-loop formation, in which case single-stranded DNA could not be fully exposed (Fig. 1B).

10.1128/mBio.00342-21.1 FIG S1 Comprehensive investigation of base editing efficiency of BE3 with hairpin sgRNAs and BH-sgRNAs at site 1. (A) Base editing efficiency using hairpin sgRNAs with different hairpin length. Predicted structures of hairpin sgRNAs are shown on the left, and bar charts on the right show on-target editing efficiency at site 1. H2 represents hairpin sgRNA with 2 nucleotides added 5′ of the spacer excluding the 4-nt loop. (B to D) Base editing efficiency using BH-sgRNAs; bubble size varied from 1 to 3 nucleotides, and position varied from 4 to 8. Predicted structures of BH-sgRNAs are shown on the left, and bar charts on the right show on-target editing efficiency at site 1. H12-B3-P5 represents BH-sgRNA with a 12-nt hairpin and 3-nt bubble, and the mismatches start from position 5. Values and error bars reflect mean ± SEM for three independent biological replicates performed on different days. Individual means and *P* values are listed in Table S1. Download FIG S1, PDF file, 2.9 MB. Copyright © 2021 Hu et al. 2021 Hu et al. https://creativecommons.org/licenses/by/4.0/ This content is distributed under the terms of the Creative Commons Attribution 4.0 International license.

10.1128/mBio.00342-21.3 TABLE S1 Means of editing efficiency and *P* values for differences in base editing under different treatment conditions at all sites evaluated in this study. Download Table S1, PDF file, 0.1 MB. Copyright © 2021 Hu et al. 2021 Hu et al. https://creativecommons.org/licenses/by/4.0/ This content is distributed under the terms of the Creative Commons Attribution 4.0 International license.

To restore the decreased base editing efficiency resulting from the extended complementary sequence, an RNA bubble formed by several unpaired consecutive nucleotides within the editing window region was introduced into hairpin sgRNA to generate a BH-sgRNA. We predicted that nucleotides within the editing window could then be exposed to deaminase in such a design (Fig. 1C).

The investigation of BH-sgRNA activity with different bubble sizes and positions showed as expected that the base editing efficiency was gradually restored with increasing bubble size, and in most cases, the restoration was most effective when the bubble was positioned at the region corresponding to the middle of the editing window (Fig. S1). Notably, the base editing efficiency was comparable to that of wild-type (WT) sgRNA when using BH-sgRNA with a 3-nt bubble positioned from positions 5 to 7 in a 12-nt hairpin (here designated H12-B3-P5) (Fig. 2B). To explore the generality of these findings, we assayed BH-sgRNA on-target editing activity at two additional sites in the E. coli genome. For all three sites, H12-B3-P5 achieved the best balance between bubble hairpin structure and base editing efficiency, and it also exhibited a similar editing window as WT-sgRNA. H12-B3-P5 was therefore chosen for further investigation.

### BH-sgRNA increases the base editing specificity of CBE in E. coli and HEK293T cells.

**FIG 3:**
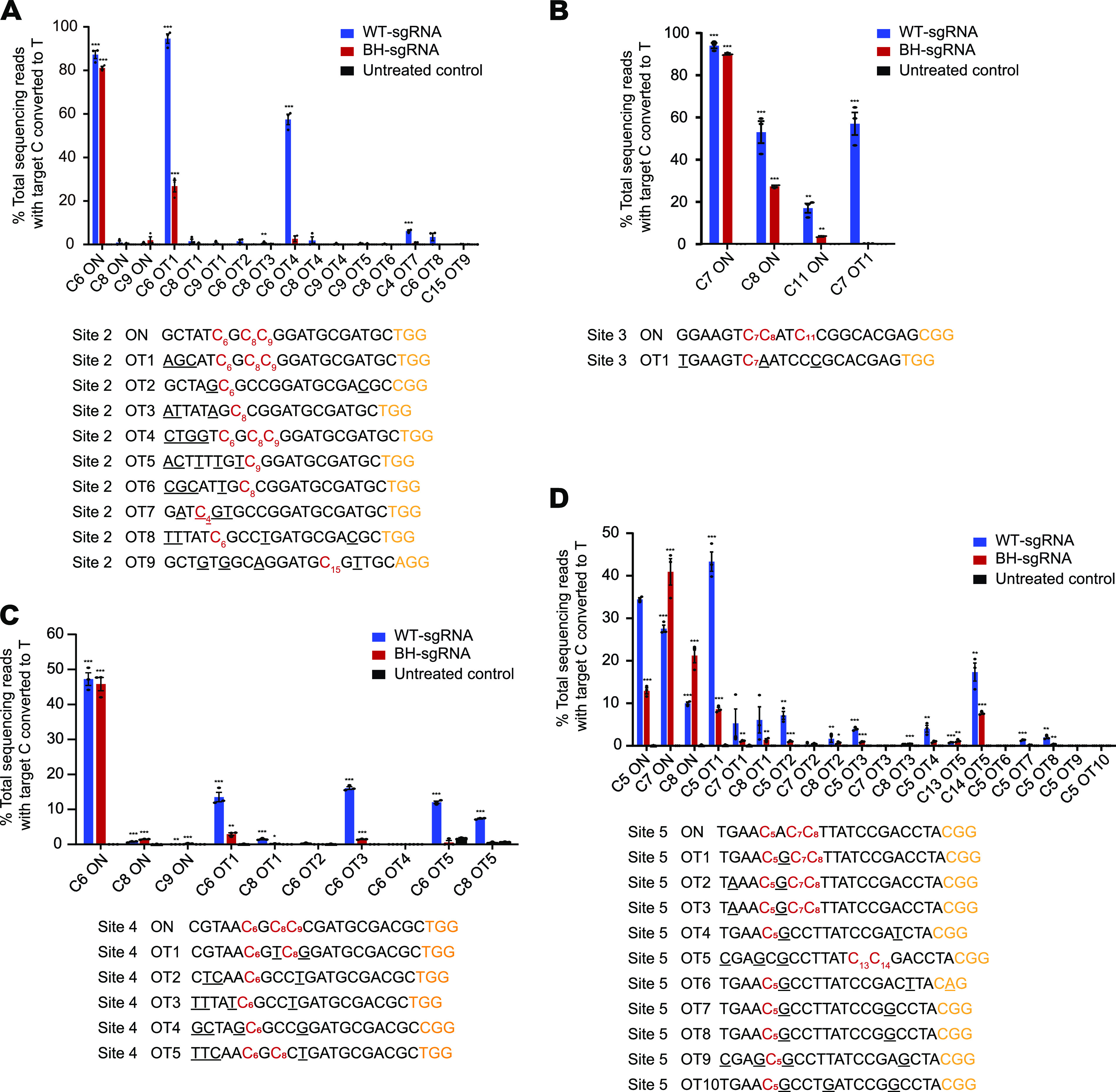
Specificity of BE3 with BH-sgRNA. On- and off-target editing associated with BE3 was assayed using deep sequencing of genomic DNA of BL21(DE3) treated with WT-sgRNAs or BH-sgRNAs at site 2 (A), site 3 (B), site 4 (C), and site 5 (D). “C[number]” refers to the “C” position in the target sequence (counting the end distal to the PAM as position 1). Values and error bars reflect mean ± SEM for three independent biological replicates performed on different days. Individual means and *P* values are listed in Table S1.

To explore the genome-wide base editing specificity of BH-sgRNA, we chose several endogenous genomic loci in E. coli to interrogate on- and off-target base editing of CBE with BH-sgRNAs. We predicted potential off-target loci using two different methods (26, 27) and chose those as representative of off-target sites. The results showed that BH-sgRNA exhibited remarkable improvement of editing specificity at all sites (Fig. 3). For site 3, there was no obvious reduction of on-target editing from an average of 94.0% ± 1.3% (mean ± SEM for *n* = 3 biological replicates) with WT-sgRNA to 90.1% ± 0.4% with BH-sgRNA BE3. Strikingly, at site 3 off-target 1 (OT1), C-to-T conversion fell from 57% ± 5.3% with WT-sgRNA to the detection limit with BH-sgRNA (Fig. 3B). We also chose site 5, which posed a formidable specificity challenge with numerous predicted repetitive off-target sites, to interrogate the specificity of BH-sgRNA. Surprisingly, BE3 BH-sgRNAs retained comparable on-target activities as WT-sgRNAs while exhibiting remarkably decreased base editing activity at all predicted off-target sites. Even at site 5 OT1, which shares high sequence similarity to the on-target site with only one base mismatch outside the seed region, an extremely challenging situation for specificity improvement, BH-sgRNA led to a 5-fold reduction in absolute off-target editing compared with that of WT-sgRNA (Fig. 3D).

**FIG 4:**
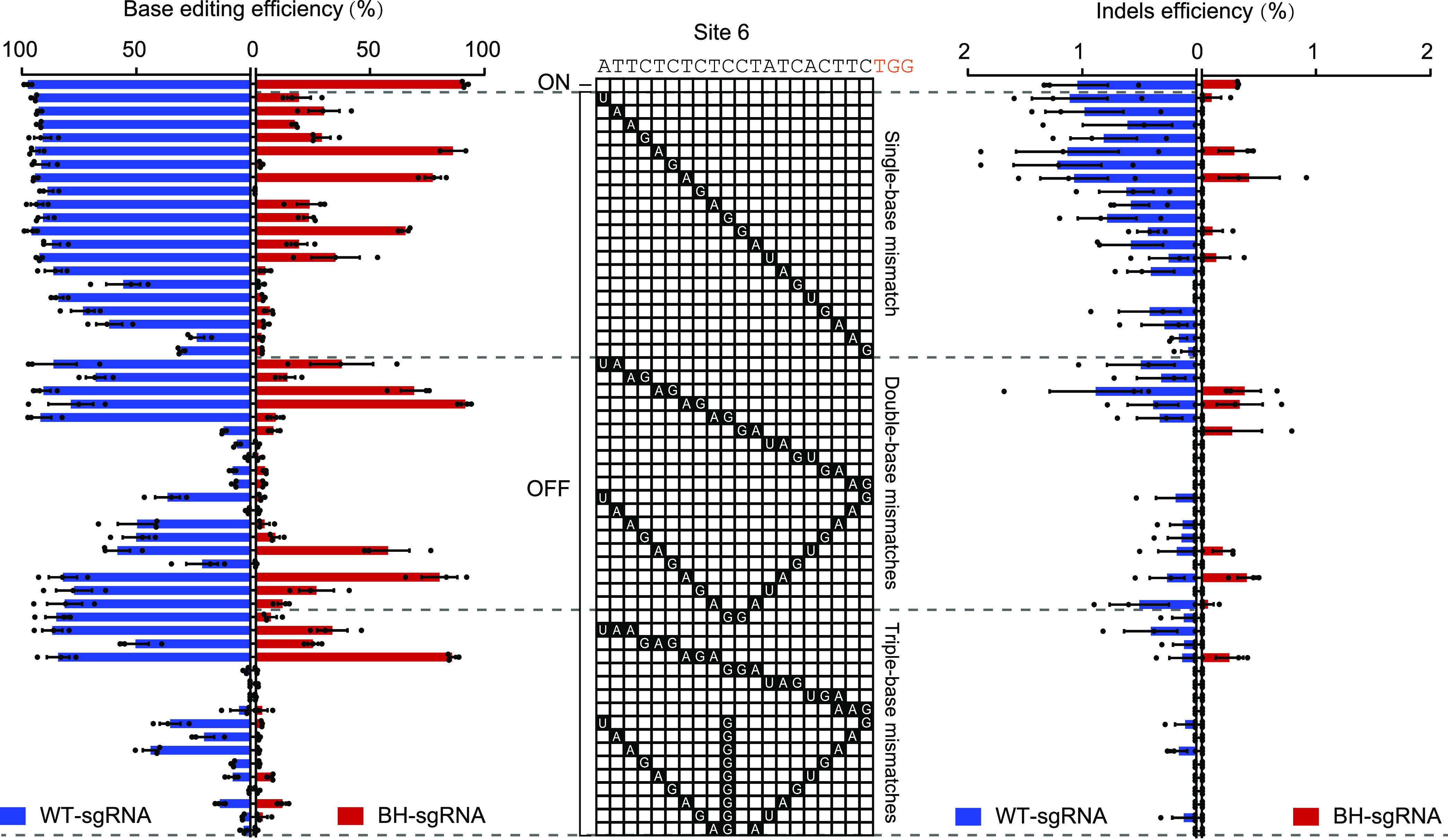
Tolerance of BE3 with BH-sgRNA for mismatched sgRNAs. Mismatched sgRNAs that differed from the on-target site by 1 to 3 nt were tested in E. coli. Base editing and indel frequencies were measured using targeted deep sequencing. Black boxes in the grids show the positions and nucleotides of mismatches.

To further understand the tolerance of BH-sgRNA for target site mismatches, we systematically mutated the guide sequence of site 6 to introduce single-, double-, and triple-base mismatches at different positions. Consistent with a previous study (11), BE3 with WT-sgRNA tolerated most of the single mismatches and double mismatches in the guide sequence, while BH-sgRNA exhibited remarkable reduction in off-target editing even with single-base mismatches located outside the seed sequence (Fig. 4). Notably, indels at the on-target site and its related off-target sites with WT-sgRNA were also found to be significantly reduced (Fig. 4).

**FIG 5:**
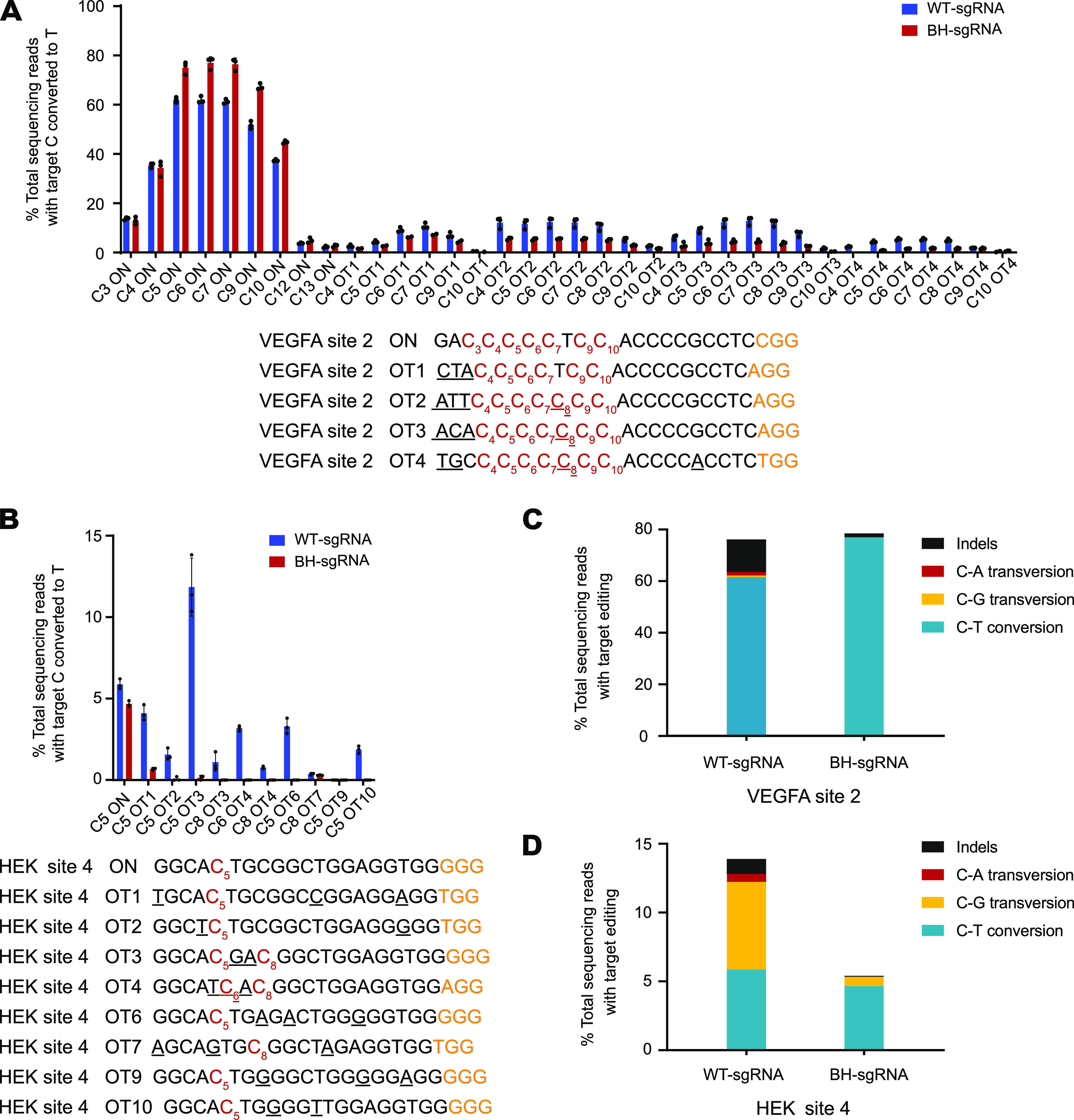
Investigation of BH-sgRNAs in human cells. (A and B) On- and off-target editing associated with BE3 were assayed using deep sequencing of genomic DNA of HEK293T cells treated with WT-sgRNAs or BH-sgRNAs at VEGFA site 2 (A) and HEK site 4 (B). (C and D) The type and ratio of on-target editing at VEGFA site 2 (C) and HEK site 4 (D). “C[number]” refers to the “C” position in the target sequence (counting the end distal to the PAM as position 1). Values and error bars reflect mean ± SEM for three independent biological replicates performed on different days. Individual means and *P* values are listed in Table S1.

Next, we chose two well-studied endogenous genomic loci (VEGFA site 2 and HEK site 4) to investigate the specificity of BH-sgRNAs in human cells (1, 10). We designed WT- and BH-sgRNAs to target VEGFA site 2 and HEK site 4. Then, plasmids encoding sgRNAs and BE3, respectively, were cotransfected into HEK293T cells. Deep sequencing results revealed that BH-sgRNAs retained considerable on-target C-to-T conversion activity (Fig. 5). Among four known off-target sites of VEGFA site 2, BH-sgRNA showed higher on-target editing efficiency than WT-sgRNA, while reducing off-target editing by 2- to 3-fold (Fig. 5A). HEK site 4 has been reported to possess many off-target sites (1); we examined the eight most frequently modified off-target sites. Compared with WT-sgRNA, although BH-sgRNA showed a slight reduction in on-target editing at HEK site 4, it dramatically decreased undesired C-to-T conversion at some off-target sites by orders of magnitude (Fig. 5B). Strikingly, we found that application of BH-sgRNAs produced fewer by-products, i.e., undesired conversion fell from 6.36% ± 0.27% to 0.67% ± 0.04% (Fig. 5C), and the indel rate at VEGFA site 2 was reduced from 12.63% ± 0.68% to 1.51% ± 0.33% (Fig. 5D). As it is known that uracil DNA glycosylase (UDG) prefers single-strand substrates to duplex substrates, we supposed that the extended sequence which paired with the edited single-strand DNA (Fig. 1C) suppresses UDG-initiated base excision repair (1).

### Whole-genome-wide off-target profiling of CBE with BH-sgRNA.

**FIG 6:**
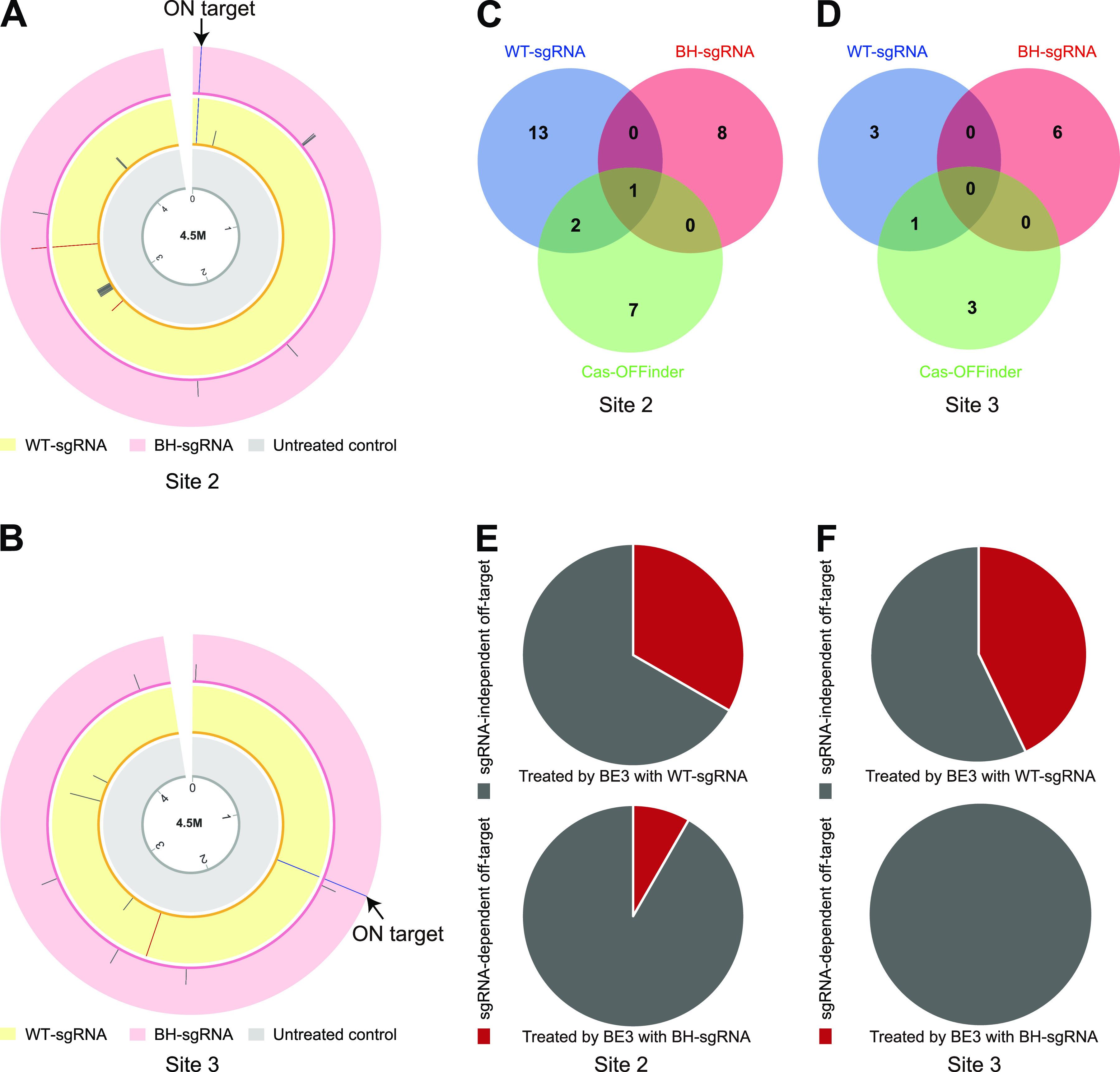
Whole-genome-wide specificity of BE3 with BH-sgRNA. (A and B) Genome-wide Circos plots represent single nucleotide variants (SNVs) obtained via whole-genome sequencing (WGS) using untreated genomic DNA (gray) and genomic DNA treated with WT-sgRNA (yellow) or with BH-sgRNA (pink) at two different sites. The blue bar, red bar, and gray bar indicate SNVs at on-target sites, the sgRNA-dependent off-target site, and the sgRNA-independent off-target site, respectively; bar height indicates mutation frequency. (C and D) Venn diagram analysis of the SNVs identified by WGS and predicted by Cas-OFFinder. SNVs were identified at site 2 (C) and site 3 (D). Mismatches between sgRNA and Cas-OFFinder predicted off-target sites no more than 4 nt. (E and F) The ratio of the sgRNA-dependent SNVs identified by WGS at site 2 (E) and site 3 (F). Positions of SNVs are listed in Table S2. Statistical qualities of WGS are listed in Table S3.

We then sought to assess whether BH-sgRNA induced additional off-target mutations in the E. coli genome besides those identified off-target sites. Whole-genome sequencing (WGS) for site 2 and site 3 was performed, and both WT- and BH-sgRNAs were interrogated. Considering that off-target editing is less likely to occur when mismatches between loci and sgRNA exceed 5 nt (11, 13), the locus sharing over 15-nt similarity with the target site is defined as the sgRNA-dependent off-target site in this study. According to whole-genome sequencing results, BH-sgRNA showed 1 sgRNA-dependent off-target edit at site 2 and no sgRNA-dependent off-target editing at site 3, while WT-sgRNA produced 3 sgRNA-dependent off-target edits at site 2 and 1 sgRNA-dependent off-target edit at site 3. Compared with WT-sgRNA, BH-sgRNA showed less sgRNA-dependent mutation than WT-sgRNA, and no unique off-target site of BH-sgRNA was found (Fig. 6A to D). In addition to sgRNA-dependent off-target mutation, we also observed a certain amount of sgRNA-independent C-to-T conversions, which may be caused by uracil DNA glycosylase inhibitor (UGI) and deaminase overexpression (11, 12, 14). Because deaminase activity at sgRNA-independent off-target sites is free from sgRNA, we did not observe obvious differences between BH-sgRNAs and WT-sgRNAs on sgRNA-independent mutation (Fig. 6).

10.1128/mBio.00342-21.4 TABLE S2 Positions of single nucleotide variants (SNVs) identified by whole-genome sequencing. Download Table S2, PDF file, 0.1 MB. Copyright © 2021 Hu et al. 2021 Hu et al. https://creativecommons.org/licenses/by/4.0/ This content is distributed under the terms of the Creative Commons Attribution 4.0 International license.

10.1128/mBio.00342-21.5 TABLE S3 Statistical quality of whole-genome sequencing analysis. Download Table S3, PDF file, 0.1 MB. Copyright © 2021 Hu et al. 2021 Hu et al. https://creativecommons.org/licenses/by/4.0/ This content is distributed under the terms of the Creative Commons Attribution 4.0 International license.

### BH-sgRNA decreases the off-target effects of ABE in E. coli.

**FIG 7:**
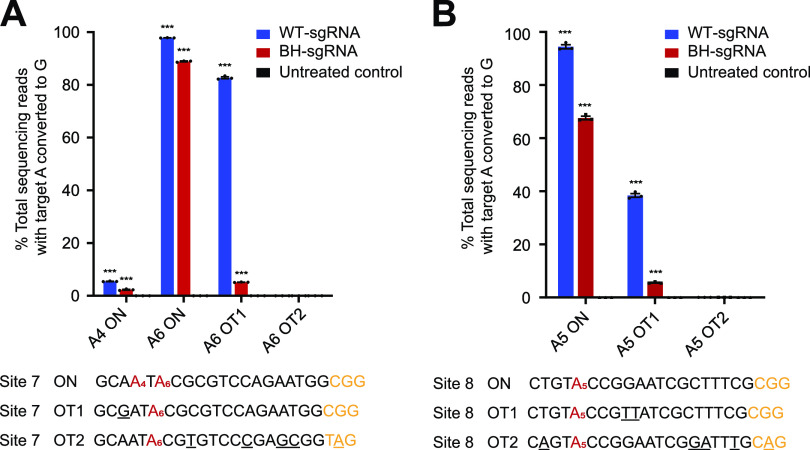
Deep sequencing investigation of BH-sgRNA specificity of ABE7.10. (A and B) On- and off-target editing associated with ABE7.10 was assayed using deep sequencing of genomic DNA from BL21(DE3) treated with WT-sgRNAs and BH-sgRNAs at site 7 in panel A and site 8 in panel B, respectively. The underlined nucleotides indicate mismatches between off-target site and on-target site. “C[number]” refers to the “C” position in the target sequence (counting the end distal to the PAM as position 1). Values and error bars reflect mean ± SEM for three independent biological replicates performed on different days. Asterisks indicate significant editing based on a comparison between the treated sample and an untreated control. *, *P* ≤ 0.05; **, *P* ≤ 0.01; ***, *P* ≤ 0.001 (Student’s two-tailed *t* test). Individual *P* values are listed in Table S1.

We also tested whether BH-sgRNA designs could be extended to adenine base editing systems. Since ABE7.10 (2) is a widely used adenine base editing system, we constructed pEcABE7.10 for the following investigation. Although sharing the same CRISPR/Cas9 system as BE3, ABE7.10 possesses a different editing window, typically from positions 4 to 7 (2) rather than positions 4 to 8 (1) for BE3 (within the protospacer counting the end distal to the protospacer-adjacent motif [PAM] as position 1). We assessed the efficiency of BH-sgRNA with a 3-nt bubble positioned from positions 4 to 6 into a 12-nt hairpin (H12-B3-P4). Since ABE7.10 is not as efficient as BE3 (28) and exhibited no editing activities at some sites in E. coli, we chose those editable sites for further investigation. BH-sgRNA showed similar editing efficiency and a similar editing window as WT-sgRNA in E. coli (Fig. S2). We then used deep sequencing to examine mutation frequencies at on-target and predicted off-target sites of site 7 and site 8. These off-target sites were predicted by Benchling (26) and Cas-OFFinder (27). Although on-target editing exhibited a slight reduction, the mutation rates showed an obvious drop at all off-target sites when using BH-sgRNA (Fig. 7).

10.1128/mBio.00342-21.2 FIG S2 Sanger sequencing investigation of BH-sgRNA efficiency for ABE7.10. On-target editing associated with ABE7.10 was assayed using Sanger sequencing of genomic DNA from BL21(DE3) treated with WT-sgRNAs and BH-sgRNAs at site 7, site 8, site 9, site 10, and site 11, respectively. The editing window is shadowed in blue, and the A-to-G conversion is indicated in a black triangle. Download FIG S2, PDF file, 0.1 MB. Copyright © 2021 Hu et al. 2021 Hu et al. https://creativecommons.org/licenses/by/4.0/ This content is distributed under the terms of the Creative Commons Attribution 4.0 International license.

## DISCUSSION

We have demonstrated a simple and practical approach to reduce the off-target effects and the by-product ratio of base editing by using BH-sgRNA. We tested numerous sgRNA secondary structures with different hairpin lengths and bubble sizes, which provides several important implications for how to design sgRNA to minimize off-target effects and by-product generation while retaining on-target editing efficiency. Our results show that BEs with BH-sgRNA possess a similar editing window and generally induce quite low or undetectable levels of base conversions and indels at off-target sites even in situations in which as few as one or two mismatches were positioned outside the seed region. Since BEs are highly modular gene editing tools, we believe our design can be combined with current and future base editing systems, such as HF-BEs (10), BE3^R33A/K34A^ (19), and ABE7.10^F148A^ (12), to further reduce off-target effects.

Although the mechanism of how BH-sgRNA increases the specificity of BEs has not been clarified, we hypothesized that it improves the base editing specificity by making the nucleotides within the editing window inaccessible to deaminase at off-target sites rather than decreasing the Cas9 binding affinity. Kocak et al. observed that even when hairpin sgRNA decreased nuclease activity by orders of magnitude, dCas9 binding was not decreased at off-target sites (22). Further biochemical experiments and structural characterization will be required to define the mechanism by which BH-sgRNA achieves its high genome-wide specificity.

In addition to hairpin length and bubble size, other characteristics such as GC content might affect BH-sgRNA activity and specificity, and it is possible that sgRNA with different secondary structures might also possess such properties. Thus, in future experiments, varied structures of sgRNA and more target sites should be investigated.

## MATERIALS AND METHODS

### Design for BH-sgRNAs.

The 12-nt reverse complement RNA sequence of the 5′ end to the spacer is added to the 5′ end of WT-sgRNA via a 5′-ACAA-3′ linker to form the hairpin sgRNA. Nucleotides positioned from positions 6 to 8 (counting the 5′ end of hairpin sgRNA as position 1) are replaced with different nucleotides to form the BH-sgRNA for CBE, and nucleotides positioned from positions 7 to 9 are replaced with different nucleotides to form the BH-sgRNA for ABE. Since noncanonical G-U base pairs can be substituted for potential G-C/A-U, we should avoid G-U base pair existence in the bubble.

### Construction of pEcABE and cloning of sgRNA.

The plasmid pEcBE3 (9) vector was used as the vector backbone for pEcABE. The rAPOBEC1 (1) was replaced by the heterodimeric wild-type TadA-TadA* (2) element and, the UGI was deleted through Gibson assembly to obtain pEcABE. For sgRNA cloning, synthesized oligonucleotides were annealed to form a dimer, which was then ligated into BsaI-digested pEcBE3 or pEcABE as previously described. PCR was performed using Phanta Max Super-Fidelity DNA polymerase (Vazyme), and Gibson assembly was performed according to a reported protocol. Oligonucleotide sequences are listed in Table S4 in the supplemental material. Bacterial strains and plasmids are listed in Tables S5 and S6, respectively.

10.1128/mBio.00342-21.6 TABLE S4 Primers used in this study. Download Table S4, PDF file, 0.1 MB. Copyright © 2021 Hu et al. 2021 Hu et al. https://creativecommons.org/licenses/by/4.0/ This content is distributed under the terms of the Creative Commons Attribution 4.0 International license.

10.1128/mBio.00342-21.7 TABLE S5 Bacterial strains used in this study. Download Table S5, PDF file, 0.1 MB. Copyright © 2021 Hu et al. 2021 Hu et al. https://creativecommons.org/licenses/by/4.0/ This content is distributed under the terms of the Creative Commons Attribution 4.0 International license.

10.1128/mBio.00342-21.8 TABLE S6 Plasmids used in this study. Download Table S6, PDF file, 0.2 MB. Copyright © 2021 Hu et al. 2021 Hu et al. https://creativecommons.org/licenses/by/4.0/ This content is distributed under the terms of the Creative Commons Attribution 4.0 International license.

### Plasmid transfection of E. coli and HEK293T cells.

Base editing was performed by transformation of E. coli BL21(DE3) competent cells with 500 ng of plasmids encoding base editors. After heat shock, transformed E. coli cells were incubated in 2× yeast extract-tryptone (YT) medium (containing 0.6 mM IPTG) at 37°C with shaking at 220 rpm for 45 min. Cells were then spread on 2× YT agar plates (containing 50 μg/ml ampicillin and 0.6 mM IPTG). The plate then was incubated at 37°C overnight (∼14 h) to obtain single colonies. HEK293T cells were obtained from the American Type Culture Collection. Cells were cultured in Dulbecco’s modified Eagle medium (DMEM) (Gibco) with 10% heat-inactivated fetal bovine serum (Gibco) and 1% penicillin-streptomycin (Gibco). Cells were maintained in a 37°C incubator with 5% CO_2_. HEK293T cells were seeded on 6-well plates (Corning) and transfected at approximately 60% confluence. Three micrograms of BE3 and 1 μg of sgRNA expression plasmids were transfected using 8 μl of Lipofectamine 3000 (ThermoFisher Scientific) per well according to the manufacturer’s protocol.

### Purification of genomic DNA.

After plasmid transfection, all colonies (>50) were collected and genomic DNA was extracted using the Bacterial Genome DNA extraction kit (Tiangen) according to the manufacturer’s instructions. Transfected cells were harvested after 48 h, and the genomic DNA was isolated using the genomic DNA isolation kit (Tiangen) according to the manufacturer’s instructions. On-target and potential off-target sites were amplified using the Phanta Max Super-Fidelity DNA polymerase (Vazyme). The primers used are listed in Table S4.

### Targeted deep sequencing.

On-target and potential off-target sites were amplified using the Phanta Max Super-Fidelity DNA polymerase (Vazyme). Amplicons were again amplified using the TruSeq HT dual index-containing primers to generate deep sequencing libraries. The libraries were sequenced using the Illumina MiniSeq at Novogene with paired-end sequencing systems. Base editing frequencies indicate the frequencies of modified target sites with at least one edit within the editing window.

### Whole-genome sequencing.

A total of 1 μg extracted genomic DNA was fragmented to around-350-bp segments using the Covaris system (ThermoFisher Scientific) and incubated with End Repair Mix (Illumina) to generate blunt ends. Then, 3′ ends were adenylated to promote precise ligation. The purified product from the A-tailing reaction was ligated with adapters to produce libraries, and then the fragments with proper size were amplified and subjected to whole-genome sequencing (WGS) using a PE150 sequencer (Illumina) at Novogene. WGS was performed at a sequencing depth of 200× to 300×. The original image data generated by the sequencing machine were converted into sequence data via base calling (Illumina pipeline CASAVA v1.8.2) and then subjected to a quality control procedure to remove unusable reads. Sequencing reads were aligned to the reference genome using BWA with default parameters. Subsequent processing, including duplicate removal, was performed using SAMtools and Picard.

### Software availability.

Cas-OFFinder is available at http://www.rgenome.net/cas-offinder; CRISPResso2 is available at https://crispresso.pinellolab.partners.org; Picard is available at http://picard.sourceforge.net.

### Data availability.

Sequencing data from this study are available in the GenBank repository under accession number PRJNA700121 (https://www.ncbi.nlm.nih.gov/bioproject/PRJNA700121).

10.1128/mBio.00342-21.9 TABLE S7 Protospacer and PAM sequences for the on- and off-target E. coli genomic loci studied in this work. Download Table S7, PDF file, 0.04 MB. Copyright © 2021 Hu et al. 2021 Hu et al. https://creativecommons.org/licenses/by/4.0/ This content is distributed under the terms of the Creative Commons Attribution 4.0 International license.
