# Supplementary material for: Improving the Precision of Base Editing by Bubble Hairpin Single Guide RNA
Source: mBio. 2021 Apr 20;12(2):e00342-21. doi: 10.1128/mBio.00342-21 (PMC8092237; doi:10.1128/mBio.00342-21)
Supplement: FIG S1 [file mBio.00342-21-sf001.pdf]

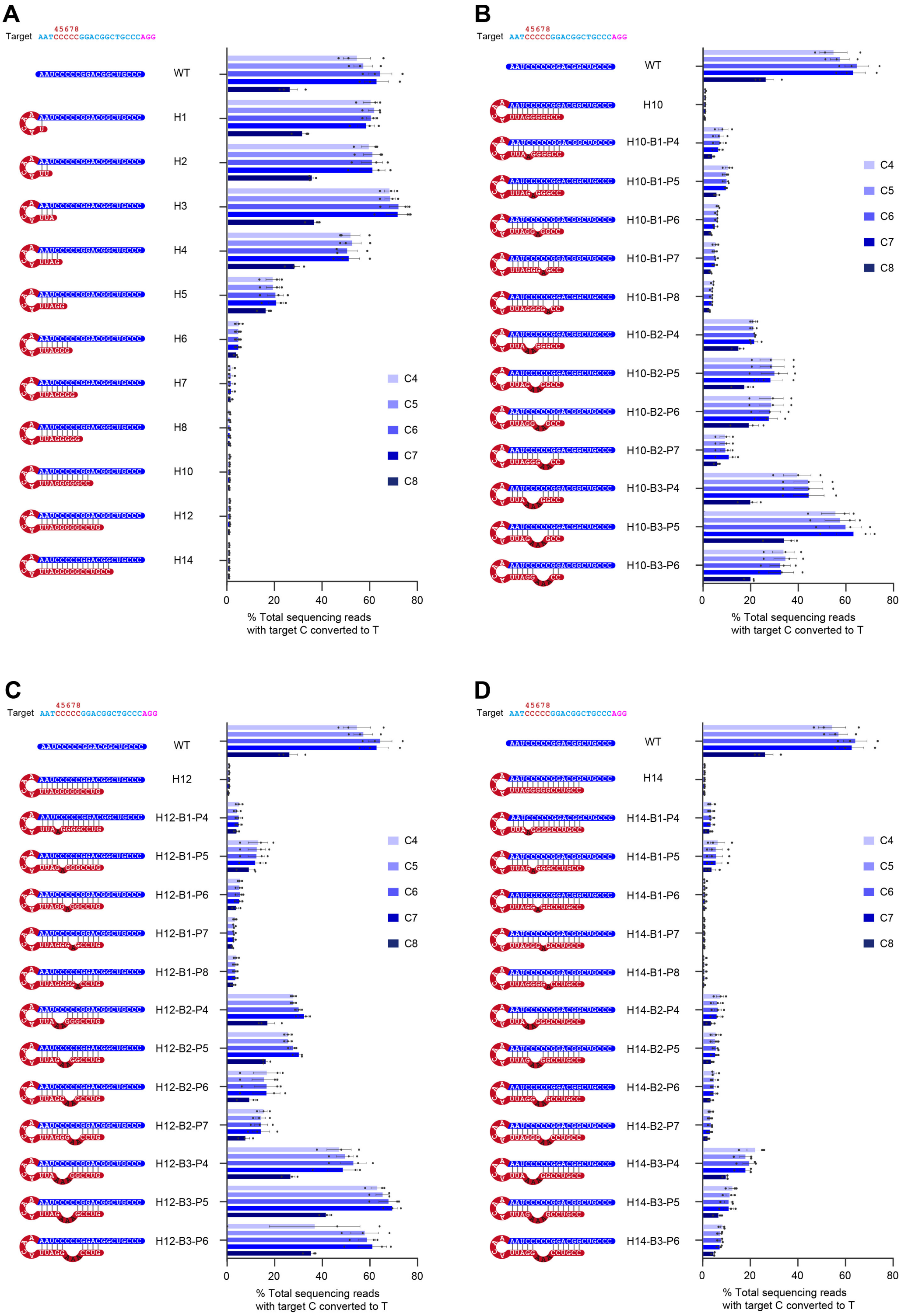

FIG S1 Comprehensive investigation of base editing efficiency of BE3 with hairpin sgRNAs and BH-sgRNAs at site 1. (A) Base editing efficiency using hairpin sgRNAs with different hairpin length. Predicted structures of hairpin sgRNAs are shown on left panel and right bar charts show on-target editing efficiency at site 1. H2 represents hairpin sgRNA with 2 nucleotides added 5' of the spacer exclude 4-nt loop. (B-D) Base editing efficiency using BH-sgRNAs, bubble size varied from 1 to 3 nucleotides and position varied from 4 to 8. Predicted structures of BH-sgRNAs are shown on left panel and right bar charts show on-target editing efficiency at site 1. H12-B3-P5 represents BH-sgRNA with 12-nt hairpin and 3-nt bubble and the mismatches start from position 5. "C_{number}" refers to the "C"-position in the target sequence (counting the end distal to the PAM as position 1). Values and error bars reflect mean ± s.e.m. of three independent biological replicates performed on different days. Individual means and *P*-values are listed in Table S1.
