# Supplementary material for: Improving the Precision of Base Editing by Bubble Hairpin Single Guide RNA
Source: mBio. 2021 Apr 20;12(2):e00342-21. doi: 10.1128/mBio.00342-21 (PMC8092237; doi:10.1128/mBio.00342-21)
Supplement: TABLE S1 [file mBio.00342-21-st001.pdf]

TABLE S1 Means of editing efficiency and *P*-values for differences in base editing under different treatment conditions at all sites evaluated in this study.

Site and editing position			Means of editing efficiency (%)			*P*-values		
			WT-sgRNA	BH-sgRNA	Untreated control	WT-sgRNA VS BH-sgRNA	WT-sgRNA VS Untreated control	BH-sgRNA VS Untreated control
CBE	Site 2	C6 ON	87.25	81.17	0.00	0.0351	0.0000	<0.000001
		C8 ON	0.90	0.38	0.00	0.5378	0.2937	0.1528
		C9 ON	0.37	1.97	0.00	0.3877	0.3739	0.2892
		C6 OT1	94.62	26.83	0.00	0.0000	0.0000	0.0006
		C8 OT1	1.47	0.31	0.00	0.3363	0.2197	0.3739
		C9 OT1	0.59	0.00	0.00	0.2905	0.2905	N/A
		C6 OT2	1.47	0.00	0.04	0.1166	0.1238	0.3739
		C8 OT3	0.65	0.29	0.00	0.3727	0.1367	0.0059
		C6 OT4	57.45	2.63	0.00	0.0000	0.0000	0.1164
		C8 OT4	1.87	0.00	0.00	0.3216	0.3216	N/A
		C9 OT4	0.23	0.00	0.00	0.3739	0.3739	N/A
		C9 OT5	0.35	0.22	0.00	0.6722	0.1496	0.3739
		C8 OT6	0.12	0.00	0.00	0.3739	0.3739	N/A
		C4 OT7	6.18	0.70	0.00	0.0006	0.0001	0.1163
		C6 OT8	3.48	0.00	0.04	0.1162	0.1197	0.3739
		C15 OT9	0.13	0.06	0.00	0.4542	0.1288	0.3739
	Site 3	C7 ON	94.02	90.11	0.00	0.0442	<0.000001	<0.000001
		C8 ON	53.06	27.35	0.00	0.0080	0.0005	<0.000001
		C11 ON	17.06	3.70	0.00	0.0039	0.0015	0.0000
		C7 OT1	57.04	0.55	0.00	0.0004	0.0004	0.0000
	Site 4	C6 ON	47.25	45.81	0.00	0.6130	0.0000	0.0000
		C8 ON	0.76	1.47	0.05	0.0080	0.0049	0.0001
		C9 ON	0.00	0.20	0.00	0.1396	N/A	0.1396
		C6 OT1	13.52	2.93	0.07	0.0016	0.0006	0.0018
		C8 OT1	1.48	0.19	0.00	0.0014	0.0006	0.0254
		C6 OT2	0.30	0.00	0.07	0.0401	0.1419	0.3739
		C6 OT3	16.09	1.46	0.00	0.0000	0.0000	0.0001
		C6 OT4	0.00	0.00	0.00	N/A	N/A	N/A
		C6 OT5	12.03	0.56	1.75	0.0001	0.0000	0.1088
		C8 OT5	7.37	0.51	0.71	0.0000	0.0000	0.2461
	Site 5	C5 ON	34.45	12.92	0.04	0.0000	<0.000001	0.0001
		C7 ON	27.55	40.92	0.00	0.0144	0.0000	0.0002
		C8 ON	9.98	21.22	0.10	0.0029	0.0000	0.0002
		C5 OT1	43.32	8.60	0.13	0.0001	0.0000	0.0000
		C7 OT1	5.27	1.15	0.08	0.2902	0.1999	0.0015
		C8 OT1	6.08	1.41	0.00	0.2055	0.1198	0.0070

		C5 OT2	7.15	1.04	0.00	0.0028	0.0015	0.0008
		C7 OT2	0.28	0.47	0.00	0.5590	0.3739	0.0034
		C8 OT2	1.67	0.70	0.00	0.3290	0.1226	0.0199
		C5 OT3	3.97	0.91	0.00	0.0003	0.0001	0.0008
		C7 OT3	0.00	0.00	0.00	N/A	N/A	N/A
		C8 OT3	0.28	0.47	0.00	0.2317	0.1162	0.0000
		C5 OT4	4.01	0.98	0.00	0.0191	0.0069	0.0026
		C13OT5	0.78	1.16	0.00	0.1443	0.0003	0.0046
		C14OT5	17.34	7.67	0.00	0.0105	0.0012	0.0000
		C5 OT6	0.00	0.00	0.00	N/A	N/A	N/A
		C5 OT7	1.33	0.17	0.00	0.0010	0.0002	0.1199
		C5 OT8	1.98	0.36	0.00	0.0055	0.0025	0.0045
		C5 OT9	0.00	0.00	0.00	N/A	N/A	N/A
		C5 OT10	0.00	0.00	0.00	N/A	N/A	N/A
	Site 6	ON	97.13	91.70	2.77	0.0140	<0.000001	<0.000001
		OT1-1	94.31	19.23	2.77	0.0001	<0.000001	0.0335
		OT1-2	92.44	30.17	2.77	0.0008	<0.000001	0.0158
		OT1-3	92.06	17.46	2.77	<0.000001	<0.000001	0.0000
		OT1-4	90.68	29.21	2.77	0.0003	0.0000	0.0024
		OT1-5	94.01	86.52	2.77	0.2243	0.0000	0.0003
		OT1-6	91.31	2.60	2.77	0.0000	0.0000	0.7269
		OT1-7	94.04	77.71	2.77	0.0106	<0.000001	0.0000
		OT1-8	88.60	0.00	2.77	0.0000	0.0000	0.0000
		OT1-9	93.14	23.88	2.77	0.0004	0.0000	0.0200
		OT1-10	90.57	23.59	2.77	0.0000	0.0000	0.0009
		OT1-11	95.59	65.78	2.77	0.0002	<0.000001	0.0000
		OT1-12	86.62	19.30	2.77	0.0002	0.0000	0.0105
		OT1-13	92.16	35.17	2.77	0.0059	<0.000001	0.0379
		OT1-14	86.02	4.49	2.77	0.0000	0.0000	0.2805
		OT1-15	55.46	2.33	2.77	0.0021	0.0021	0.6500
		OT1-16	83.80	3.62	2.77	0.0000	0.0000	0.1183
		OT1-17	73.11	6.50	2.77	0.0002	0.0002	0.0387
		OT1-18	61.58	4.51	2.77	0.0006	0.0005	0.1157
		OT1-19	23.30	2.87	2.77	0.0034	0.0033	0.8560
		OT1-20	30.32	3.13	2.77	0.0000	0.0000	0.0631
		OT2-1	85.94	37.90	2.77	0.0483	0.0012	0.0634
		OT2-3	67.51	14.19	2.77	0.0006	0.0001	0.0283
		OT2-5	90.34	69.60	2.77	0.0363	0.0000	0.0004
		OT2-7	78.37	91.92	2.77	0.2474	0.0016	0.0000
		OT2-9	91.68	9.00	2.77	0.0001	0.0000	0.0359
		OT2-11	11.61	8.07	2.77	0.0992	0.0002	0.0262
		OT2-13	5.77	1.15	2.77	0.0105	0.0275	0.0387
		OT2-15	1.19	1.51	2.77	0.7950	0.0467	0.2797

		OT2-17	7.61	4.40	2.77	0.0281	0.0048	0.0239
		OT2-19	6.80	3.82	2.77	0.0220	0.0041	0.0979
		OT2-1S	36.14	2.87	2.77	0.0036	0.0035	0.9067
		OT2-2S	1.06	1.64	2.77	0.3872	0.0309	0.0280
		OT2-3S	49.36	4.19	2.77	0.0067	0.0053	0.5513
		OT2-4S	49.84	8.94	2.77	0.0026	0.0013	0.0316
		OT2-5S	58.01	58.18	2.77	0.9882	0.0005	0.0041
		OT2-6S	20.99	0.37	2.77	0.0411	0.0584	0.0012
		OT2-7S	81.82	80.64	2.77	0.9116	0.0002	0.0006
		OT2-8S	76.95	26.90	2.77	0.0101	0.0007	0.0339
		OT2-9S	81.09	12.07	2.77	0.0010	0.0005	0.0123
		OT2-10S	84.70	6.97	2.77	0.0001	0.0001	0.1732
		OT3-1	86.45	33.84	2.77	0.0027	0.0000	0.0094
		OT3-4	50.09	25.33	2.77	0.0160	0.0012	0.0006
		OT3-7	83.93	86.12	2.77	0.6927	0.0001	<0.000001
		OT3-10	1.81	0.67	2.77	0.2070	0.2362	0.0046
		OT3-13	0.00	0.93	2.77	0.1164	0.0000	0.0184
		OT3-16	0.00	0.25	2.77	0.1948	0.0000	0.0002
		OT3-18	4.81	3.19	2.77	0.7367	0.6239	0.8673
		OT3-1S	34.99	2.96	2.77	0.0022	0.0021	0.3997
		OT3-2S	20.11	1.70	2.77	0.0150	0.0181	0.0432
		OT3-3S	43.49	1.59	2.77	0.0002	0.0003	0.0193
		OT3-4S	7.44	1.89	2.77	0.0003	0.0006	0.0096
		OT3-5S	7.62	6.95	2.77	0.7475	0.0482	0.0095
		OT3-6S	0.31	1.06	2.77	0.3074	0.0003	0.0538
		OT3-7S	13.23	12.06	2.77	0.5830	0.0013	0.0033
		OT3-8S	3.11	3.42	2.77	0.8854	0.3322	0.7605
		OT3-9S	2.81	0.42	2.77	0.0745	0.9671	0.0060
ABE	Site 7	C4 ON	5.47	2.31	0.00	0.0000	<0.000001	0.0001
		C6 ON	97.83	88.89	0.00	<0.000001	<0.000001	<0.000001
		C6 OT1	82.70	5.15	0.00	<0.000001	<0.000001	<0.000001
		C6 OT2	0.00	0.00	0.00	N/A	N/A	N/A
	Site 8	C5 ON	94.45	67.60	0.00	0.0000	<0.000001	<0.000001
		C5 OT1	38.43	5.55	0.00	0.0000	<0.000001	0.0000
		C5 OT2	0.00	0.00	0.00	N/A	N/A	N/A
HEK293T CBE	VEGFA Site 2	C3 ON	13.78	12.91	/	0.402411	/	/
		C4 ON	35.46	34.34	/	0.586811	/	/
		C5 ON	61.97	75.08	/	0.001170	/	/
		C6 ON	61.97	76.94	/	0.000797	/	/
		C7 ON	61.31	76.49	/	0.000524	/	/
		C9 ON	51.68	67.29	/	0.000186	/	/
		C10 ON	37.40	44.81	/	0.000067	/	/
		C12 ON	3.75	4.90	/	0.147664	/	/

		C13 ON	2.29	2.72	/	0.312417	/	/
		C4 OT1	2.60	1.09	/	0.072070	/	/
		C5 OT1	4.35	1.80	/	0.054737	/	/
		C6 OT1	9.18	4.21	/	0.084440	/	/
		C7 OT1	10.78	4.86	/	0.078683	/	/
		C9 OT1	7.04	2.95	/	0.064779	/	/
		C10 OT1	0.55	0.09	/	0.026349	/	/
		C4 OT2	12.11	5.62	/	0.007618	/	/
		C5 OT2	11.61	5.41	/	0.006096	/	/
		C6 OT2	12.37	5.57	/	0.004572	/	/
		C7 OT2	12.20	5.56	/	0.003504	/	/
		C8 OT2	10.66	5.15	/	0.004962	/	/
		C9 OT2	5.45	2.91	/	0.003941	/	/
		C10 OT2	2.70	1.66	/	0.007340	/	/
		C4 OT3	6.19	3.02	/	0.019812	/	/
		C5 OT3	9.19	4.05	/	0.005163	/	/
		C6 OT3	12.27	4.51	/	0.002210	/	/
		C7 OT3	12.88	4.51	/	0.001357	/	/
		C8 OT3	12.10	3.78	/	0.000720	/	/
		C9 OT3	7.70	1.99	/	0.004184	/	/
		C10 OT3	1.60	0.27	/	0.009052	/	/
		C4 OT4	2.32	0.00	/	0.000205	/	/
		C5 OT4	4.38	0.82	/	0.000187	/	/
		C6 OT4	5.39	1.59	/	0.000199	/	/
		C7 OT4	5.39	1.77	/	0.000269	/	/
		C8 OT4	4.97	1.68	/	0.000856	/	/
		C9 OT4	1.86	1.68	/	0.371976	/	/
		C10 OT4	0.40	0.60	/	0.347429	/	/
	HEK Site 4	C5 ON	5.86	4.66	/	0.004151	/	/
		C5 OT1	4.10	0.68	/	0.000241	/	/
		C5 OT2	1.57	0.07	/	0.002216	/	/
		C5 OT3	11.82	0.15	/	0.000346	/	/
		C8 OT3	1.10	0.00	/	0.027452	/	/
		C6 OT4	3.17	0.00	/	0.000004	/	/
		C8 OT4	0.77	0.00	/	0.000076	/	/
		C5 OT6	3.29	0.00	/	0.000274	/	/
		C8 OT7	0.37	0.29	/	0.079788	/	/
		C5 OT9	0.00	0.00	/	N/A	/	/
		C5 OT10	1.86	0.00	/	0.000138	/	/
