# Supplementary material for: Improving the Precision of Base Editing by Bubble Hairpin Single Guide RNA
Source: mBio. 2021 Apr 20;12(2):e00342-21. doi: 10.1128/mBio.00342-21 (PMC8092237; doi:10.1128/mBio.00342-21)
Supplement: TABLE S2 [file mBio.00342-21-st002.pdf]

TABLE S2 Positions of SNVs identified by whole-genome sequencing.

Sample name	Chromosome	Position	Reference	Alter	Sequence
BE3-site 3-#1	NC_012971.20	1419216	G	A	CTCGTGCCGGAT**G**GACTTCC
	NC_012971.19	1419217	G	A	CTCGTGCCGGATG**G**ACTTCC
	NC_012971.18	2511689	G	A	GTGCGGGATT**G**ACTTCATGCAGCGGTAGAG
	NC_012971.17	2760158	G	A	CGGCGATAAA**G**AGATTATGCGTCGTATGGA
	NC_012971.16	3603545	C	T	GCGCACGGTG**C**ATCTTCACACTGATGACAG
BE3-site 3-#2	NC_012971.15	1419217	G	A	CTCGTGCCGGATG**G**ACTTCC
	NC_012971.14	2511689	G	A	GTGCGGGATT**G**ACTTCATGCAGCGGTAGAG
	NC_012971.13	3603545	C	T	GCGCACGGTG**C**ATCTTCACACTGATGACAG
	NC_012971.12	3758599	G	A	AGACGGCTGG**G**AGCAACATATTACCGCCC
BE3-site 3-#3	NC_012971.11	1419216	G	A	CTCGTGCCGGAT**G**GACTTCC
	NC_012971.10	1419217	G	A	CTCGTGCCGGATG**G**ACTTCC
	NC_012971.9	2511689	G	A	GTGCGGGATT**G**ACTTCATGCAGCGGTAGAG
BH BE3-site 3-#1	NC_012971.8	1419217	G	A	CTCGTGCCGGATG**G**ACTTCC
	NC_012971.7	2658162	G	A	TCAAGTAAGA**G**ATGGTGCATCCGGGAGGAT
BH BE3-site 3-#2	NC_012971.6	1419217	G	A	CTCGTGCCGGATG**G**ACTTCC
	NC_012971.5	2298808	G	A	TAACCGCAAT**G**ACATGGCAACCCGCCGCCA
BH BE3-site 3-#3	NC_012971.4	27020	C	T	AGCCGGTACA**C**TGGTGCGTTGACTGCCGTT
	NC_012971.3	1419217	G	A	CTCGTGCCGGATG**G**ACTTCC
	NC_012971.2	1454792	G	A	ATCATCTGGG**G**ATTATTGACTAGCGCATTTA
	NC_012971.1	3139263	G	A	TAGCGGCAAT**G**GCATCGACGTTATCTACGC
	NC_012971.0	4299095	C	T	AAGGCACAGA**C**CACCCAAGCGGGAATAGC
BE3-site 2-#1	NC_012971.1	38798	C	T	GCGACCTGGT**C**GCCGGATGCGATGCTGGC
	NC_012971.2	38888	C	T	CGACAGCTAT**C**GCCGGATGCGATGCTGGC
	NC_012971.2	3364423	G	A	GCGCCAGCAT**C**GCCGGATGCGATGCTGGC
BE3-site 2-#2	NC_012971.2	38798	C	T	GCGACCTGGT**C**GCCGGATGCGATGCTGGC
	NC_012971.2	38888	C	T	CGACAGCTAT**C**GCCGGATGCGATGCTGGC
	NC_012971.2	167433	A	C	GGGTGAGGGC**A**TCAGCGCGCACGTTCACC
	NC_012971.2	2873180	C	T	TTTAT**C***GCCTGATGCGACGC*TGG
	NC_012971.2	3022755	C	T	GCAGATCGTT**C**TCTGCCCTCATATTGGCCCA
	NC_012971.2	3022757	C	T	AGATCGTTCT**C**TGCCCTCATATTGGCCCAGC
	NC_012971.2	3022896	C	T	TATTGATTAC**C**TACGGCGGCGGCAGCGTGA
	NC_012971.2	3023055	C	T	AAGTGACTTT**C**CTGCTGGCGGTTGGCGGCG
	NC_012971.2	3023103	C	T	CCAAATTTAT**C**GCCGCAGCGGCTAACTATCC
	NC_012971.2	3023133	C	T	CGGAAAATAT**C**GATCCGTGGCACATTCTGC
	NC_012971.2	3023405	C	T	CAGTATGTTA**C**CAAACCGGTTGATGCCAAA
	NC_012971.2	3023406	C	T	AGTATGTTAC**C**AAACCGGTTGATGCCAAAAT
	NC_012971.2	3025054	C	T	TCATCTATAT**C**CGGGATAAACGCAACGGAG
	NC_012971.2	3026475	C	T	GGGTCGCCAT**C**GGCGTATGTTTCGTCAAC
	NC_012971.2	3364423	G	A	GCGCCAGCAT**C**GCCGGATGCGATGCTGG
	NC_012971.2	38798	C	T	GCGACCTGGT**C**GCCGGATGCGATGCTGGC

BE3-site 2-#3	NC_012971.2	38888	C	T	CGACAGCTAT**C**GCCGGATGCGATGCTGGC
	NC_012971.2	3364423	G	A	GCGCCAGCAT**C**GCCGGATGCGATGCTGGC
	NC_012971.2	4004867	C	T	TCTTGCCGGT**C**AGGCGATAACTGAAGTAAT
	NC_012971.2	4012165	C	T	TGAGAGATAT**C**AGAAAGCAGACGCTGCTGA
BH BE3-site 2-#1	NC_012971.2	38888	C	T	CGACAGCTAT**C**GCCGGATGCGATGCTGGC
	NC_012971.2	3364423	G	A	GCGCCAGCAT**C**GCCGGATGCGATGCTGGC
BH BE3-site 2-#2	NC_012971.2	38888	C	T	CGACAGCTAT**C**GCCGGATGCGATGCTGGC
	NC_012971.2	634018	C	T	ACGCCTTTTT**C**AGTCAGGGCTTGCTGAGAAA
	NC_012971.2	640893	C	T	CACCGTCTTT**C**CAGACGGCAATTAAACTATT
	NC_012971.2	645401	C	T	CCGATTTTCT**C**CGGCAGCAGCTCCGGGATC
	NC_012971.2	1752187	Indels		TATGATCTGG**CAGACAACATGGGAGAGACA**
	NC_012971.2	1752193	A	T	CTGGCAGACA**A**CATGGGAGAGACATCATGT
	NC_012971.2	2247744	G	A	ATGGTGATAT**C**CACCAACGGAAAAGCTTCG
BH BE3-site 2-#3	NC_012971.2	38888	C	T	CGACAGCTAT**C**GCCGGATGCGATGCTGGC
	NC_012971.2	167433	A	C	GGGTGAGGGC**A**TCAGCGCGCACGTTCACC
	NC_012971.2	3530791	G	A	TGCTCAATCA**G**AAAATTCAAAACCCTAATCC
WT	/	/	/	/	/

Note: SNVs predicted by Cas-OFFinder are shadowed in pink and the conversion nucleotides are shown in bold. # represents independent biological replicates.
