# Supplementary material for: Improving the Precision of Base Editing by Bubble Hairpin Single Guide RNA
Source: mBio. 2021 Apr 20;12(2):e00342-21. doi: 10.1128/mBio.00342-21 (PMC8092237; doi:10.1128/mBio.00342-21)
Supplement: TABLE S3 [file mBio.00342-21-st003.pdf]

TABLE S3 Statistics quality of whole-genome sequencing analysis.

Sample name	Clean base (Mb)	Mapping rate (%)	Insert size (bp)	Coverage (>Q30)	Average depth (×)
BE3-site 2-#1	1523.71	93.27	350	90.75	259.45
BE3-site 2-#2	1709.9	94.96	350	90.64	305.75
BE3-site 2-#3	1565.56	94.56	350	89.83	281.49
BE3-site 3-#1	1801.18	97.61	350	91.97	313.79
BE3-site 3-#2	1522.03	99.18	350	91.36	276.57
BE3-site 3-#3	1444.9	97.99	350	91.19	255.36
BH BE3-site 2-#1	1545.69	93.88	350	89.94	273.17
BH BE3-site 2-#2	1175.43	94.71	350	91.34	217.98
BH BE3-site 2-#3	1726.48	94.31	350	90.34	298.07
BH BE3-site 3-#1	1149.47	97.28	350	90.98	200.38
BH BE3-site 3-#2	1722.81	99.43	350	91.78	310.03
BH BE3-site 3-#3	1161.49	98.63	350	91.29	208.11
WT	1893.4	99.65	350	90.38	346.95

Note: # represents independent biological replicates.
