# Supplementary material for: Improving the Precision of Base Editing by Bubble Hairpin Single Guide RNA
Source: mBio. 2021 Apr 20;12(2):e00342-21. doi: 10.1128/mBio.00342-21 (PMC8092237; doi:10.1128/mBio.00342-21)
Supplement: FIG S2 [file mBio.00342-21-sf002.pdf]

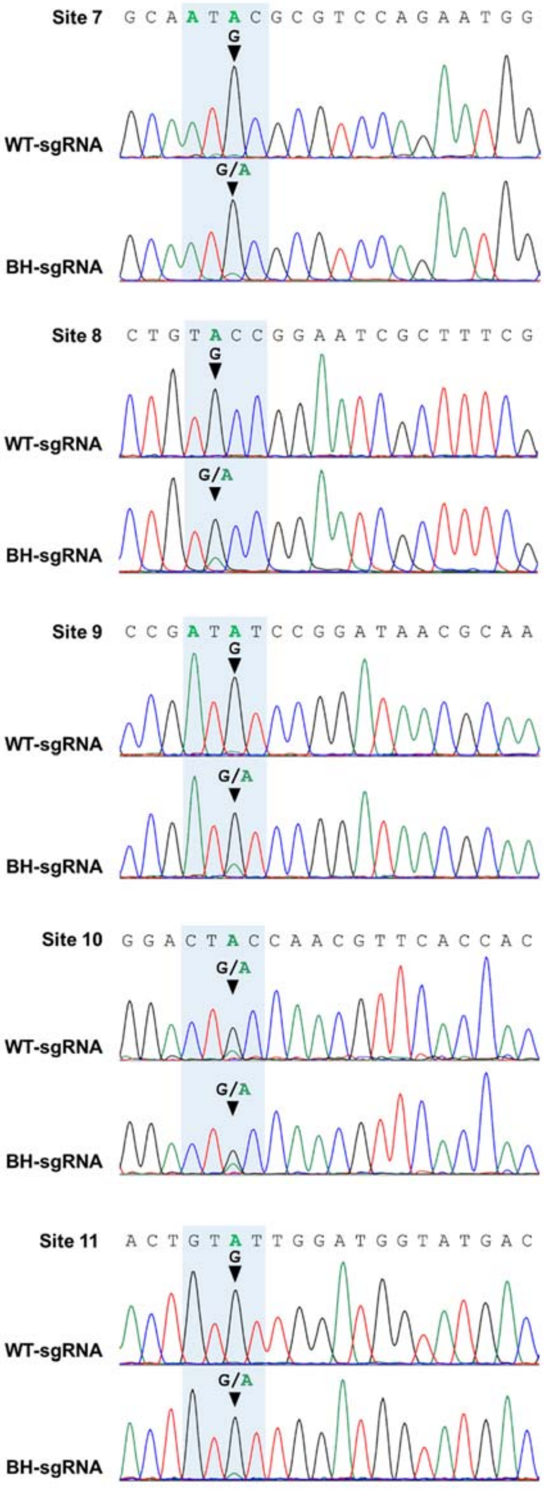

FIG S2 Sanger sequencing investigation of BH-sgRNAs efficiency for ABE7.10. On-target editing associated with ABE7.10 was assayed using Sanger sequencing of genomic DNA from BL21(DE3) treated with WT-sgRNAs and BH-sgRNAs at site 7, site 8, site 9, site 10 ,and site 11, respectively. The editing window is shadowed in blue, and the A-to-G conversion is indicated in black triangle.
