# Supplementary material for: Improving the Precision of Base Editing by Bubble Hairpin Single Guide RNA
Source: mBio. 2021 Apr 20;12(2):e00342-21. doi: 10.1128/mBio.00342-21 (PMC8092237; doi:10.1128/mBio.00342-21)
Supplement: TABLE S4 [file mBio.00342-21-st004.pdf]

TABLE S4 Primers used in this study.

Primer name	Sequence (5'-3')
**Site 1 BE3 sgRNA cloning**	
Site 1 WT-sgRNA F	GCACACAAAATCCCCCGGACGGCTGCCC
Site 1 WT-sgRNA R	AAACGGGCAGCCGTCCGGGGGATTTTGT
Site 1 Hp-sgRNA H1 F	GCACTACAAAATCCCCCGGACGGCTGCCC
Site 1 Hp-sgRNA H1 R	AAACGGGCAGCCGTCCGGGGGATTTTGTA
Site 1 Hp-sgRNA H2 F	GCACTTACAAAATCCCCCGGACGGCTGCCC
Site 1 Hp-sgRNA H2 R	AAACGGGCAGCCGTCCGGGGGATTTTGTAA
Site 1 Hp-sgRNA H3 F	GCACATTACAAAATCCCCCGGACGGCTGCCC
Site 1 Hp-sgRNA H3 R	AAACGGGCAGCCGTCCGGGGGATTTTGTAAT
Site 1 Hp-sgRNA H4 F	GCACGATTACAAAATCCCCCGGACGGCTGCCC
Site 1 Hp-sgRNA H4 R	AAACGGGCAGCCGTCCGGGGGATTTTGTAATC
Site 1 Hp-sgRNA H5 F	GCACGGATTACAAAATCCCCCGGACGGCTGCCC
Site 1 Hp-sgRNA H5 R	AAACGGGCAGCCGTCCGGGGGATTTTGTAATCC
Site 1 Hp-sgRNA H6 F	GCACGGGATTACAAAATCCCCCGGACGGCTGCCC
Site 1 Hp-sgRNA H6 R	AAACGGGCAGCCGTCCGGGGGATTTTGTAATCCC
Site 1 Hp-sgRNA H7 F	GCACGGGGATTACAAAATCCCCCGGACGGCTGCCC
Site 1 Hp-sgRNA H7 R	AAACGGGCAGCCGTCCGGGGGATTTTGTAATCCCC
Site 1 Hp-sgRNA H8 F	GCACGGGGGATTACAAAATCCCCCGGACGGCTGCCC
Site 1 Hp-sgRNA H8 R	AAACGGGCAGCCGTCCGGGGGATTTTGTAATCCCCC
Site 1 Hp-sgRNA H10 F	GCACCCGGGGGATTACAAAATCCCCCGGACGGCTGCCC
Site 1 Hp-sgRNA H10 R	AAACGGGCAGCCGTCCGGGGGATTTTGTAATCCCCCGG
Site 1 Hp-sgRNA H12 F	GCACGTCCGGGGGATTACAAAATCCCCCGGACGGCTGCCC
Site 1 Hp-sgRNA H12 R	AAACGGGCAGCCGTCCGGGGGATTTTGTAATCCCCCGGAC
Site 1 Hp-sgRNA H14 F	GCACCCGTCCGGGGGATTACAAAATCCCCCGGACGGCTGCCC
Site 1 Hp-sgRNA H14 R	AAACGGGCAGCCGTCCGGGGGATTTTGTAATCCCCCGGACGG
Site 1 BH-sgRNA H10-B1-P4 F	GCACCCGGGGTATTACAAAATCCCCCGGACGGCTGCCC
Site 1 BH-sgRNA H10-B1-P4 R	AAACGGGCAGCCGTCCGGGGGATTTTGTAATACCCCGG
Site 1 BH-sgRNA H10-B1-P5 F	GCACCCGGGTGATTACAAAATCCCCCGGACGGCTGCCC
Site 1 BH-sgRNA H10-B1-P5 R	AAACGGGCAGCCGTCCGGGGGATTTTGTAATCACCCGG
Site 1 BH-sgRNA H10-B1-P6 F	GCACCCGGTGGATTACAAAATCCCCCGGACGGCTGCCC
Site 1 BH-sgRNA H10-B1-P6 R	AAACGGGCAGCCGTCCGGGGGATTTTGTAATCCACCGG
Site 1 BH-sgRNA H10-B1-P7 F	GCACCCGTGGGATTACAAAATCCCCCGGACGGCTGCCC
Site 1 BH-sgRNA H10-B1-P7 R	AAACGGGCAGCCGTCCGGGGGATTTTGTAATCCCACGG
Site 1 BH-sgRNA H10-B1-P8 F	GCACCCTGGGGATTACAAAATCCCCCGGACGGCTGCCC
Site 1 BH-sgRNA H10-B1-P8 R	AAACGGGCAGCCGTCCGGGGGATTTTGTAATCCCCAGG
Site 1 BH-sgRNA H10-B2-P4 F	GCACCCGGGTTATTACAAAATCCCCCGGACGGCTGCCC
Site 1 BH-sgRNA H10-B2-P4 R	AAACGGGCAGCCGTCCGGGGGATTTTGTAATAACCCGG
Site 1 BH-sgRNA H10-B2-P5 F	GCACCCGGTTGATTACAAAATCCCCCGGACGGCTGCCC
Site 1 BH-sgRNA H10-B2-P5 R	AAACGGGCAGCCGTCCGGGGGATTTTGTAATCAACCGG
Site 1 BH-sgRNA H10-B2-P6 F	GCACCCGTTGGATTACAAAATCCCCCGGACGGCTGCCC
Site 1 BH-sgRNA H10-B2-P6 R	AAACGGGCAGCCGTCCGGGGGATTTTGTAATCCAACGG

Site 1 BH-sgRNA H10-B2-P7 F	GCACCCTTGGGATTACAAAATCCCCCGGACGGCTGCCC
Site 1 BH-sgRNA H10-B2-P7 R	AAACGGGCAGCCGTCCGGGGGATTTTGTAATCCCAAGG
Site 1 BH-sgRNA H10-B3-P4 F	GCACCCGGTTTATTACAAAATCCCCCGGACGGCTGCCC
Site 1 BH-sgRNA H10-B3-P4 R	AAACGGGCAGCCGTCCGGGGGATTTTGTAATAAACCGG
Site 1 BH-sgRNA H10-B3-P5 F	GCACCCGTTTGATTACAAAATCCCCCGGACGGCTGCCC
Site 1 BH-sgRNA H10-B3-P5 R	AAACGGGCAGCCGTCCGGGGGATTTTGTAATCAAACGG
Site 1 BH-sgRNA H10-B3-P6 F	GCACCCTTTGGATTACAAAATCCCCCGGACGGCTGCCC
Site 1 BH-sgRNA H10-B3-P6 R	AAACGGGCAGCCGTCCGGGGGATTTTGTAATCCAAAGG
Site 1 BH-sgRNA H12-B1-P4 F	GCACGTCCGGGGTATTACAAAATCCCCCGGACGGCTGCCC
Site 1 BH-sgRNA H12-B1-P4 R	AAACGGGCAGCCGTCCGGGGGATTTTGTAATACCCCGGAC
Site 1 BH-sgRNA H12-B1-P5 F	GCACGTCCGGGTGATTACAAAATCCCCCGGACGGCTGCCC
Site 1 BH-sgRNA H12-B1-P5 R	AAACGGGCAGCCGTCCGGGGGATTTTGTAATCACCCGGAC
Site 1 BH-sgRNA H12-B1-P6 F	GCACGTCCGGTGGATTACAAAATCCCCCGGACGGCTGCCC
Site 1 BH-sgRNA H12-B1-P6 R	AAACGGGCAGCCGTCCGGGGGATTTTGTAATCCACCGGAC
Site 1 BH-sgRNA H12-B1-P7 F	GCACGTCCGTGGGATTACAAAATCCCCCGGACGGCTGCCC
Site 1 BH-sgRNA H12-B1-P7 R	AAACGGGCAGCCGTCCGGGGGATTTTGTAATCCCACGGAC
Site 1 BH-sgRNA H12-B1-P8 F	GCACGTCCTGGGGATTACAAAATCCCCCGGACGGCTGCCC
Site 1 BH-sgRNA H12-B1-P8 R	AAACGGGCAGCCGTCCGGGGGATTTTGTAATCCCCAGGAC
Site 1 BH-sgRNA H12-B2-P4 F	GCACGTCCGGGTTATTACAAAATCCCCCGGACGGCTGCCC
Site 1 BH-sgRNA H12-B2-P4 R	AAACGGGCAGCCGTCCGGGGGATTTTGTAATAACCCGGAC
Site 1 BH-sgRNA H12-B2-P5 F	GCACGTCCGGTTGATTACAAAATCCCCCGGACGGCTGCCC
Site 1 BH-sgRNA H12-B2-P5 R	AAACGGGCAGCCGTCCGGGGGATTTTGTAATCAACCGGAC
Site 1 BH-sgRNA H12-B2-P6 F	GCACGTCCGTTGGATTACAAAATCCCCCGGACGGCTGCCC
Site 1 BH-sgRNA H12-B2-P6 R	AAACGGGCAGCCGTCCGGGGGATTTTGTAATCCAACGGAC
Site 1 BH-sgRNA H12-B2-P7 F	GCACGTCCTTGGGATTACAAAATCCCCCGGACGGCTGCCC
Site 1 BH-sgRNA H12-B2-P7 R	AAACGGGCAGCCGTCCGGGGGATTTTGTAATCCCAAGGAC
Site 1 BH-sgRNA H12-B3-P4 F	GCACGTCCGGTTTATTACAAAATCCCCCGGACGGCTGCCC
Site 1 BH-sgRNA H12-B3-P4 R	AAACGGGCAGCCGTCCGGGGGATTTTGTAATAAACCGGAC
Site 1 BH-sgRNA H12-B3-P5 F	GCACGTCCGTTTGATTACAAAATCCCCCGGACGGCTGCCC
Site 1 BH-sgRNA H12-B3-P5 R	AAACGGGCAGCCGTCCGGGGGATTTTGTAATCAAACGGAC
Site 1 BH-sgRNA H12-B3-P6 F	GCACGTCCTTTGGATTACAAAATCCCCCGGACGGCTGCCC
Site 1 BH-sgRNA H12-B3-P6 R	AAACGGGCAGCCGTCCGGGGGATTTTGTAATCCAAAGGAC
Site 1 BH-sgRNA H14-B1-P4 F	GCACCCGTCCGGGGTATTACAAAATCCCCCGGACGGCTGCCC
Site 1 BH-sgRNA H14-B1-P4 R	AAACGGGCAGCCGTCCGGGGGATTTTGTAATACCCCGGACGG
Site 1 BH-sgRNA H14-B1-P5 F	GCACCCGTCCGGGTGATTACAAAATCCCCCGGACGGCTGCCC
Site 1 BH-sgRNA H14-B1-P5 R	AAACGGGCAGCCGTCCGGGGGATTTTGTAATCACCCGGACGG
Site 1 BH-sgRNA H14-B1-P6 F	GCACCCGTCCGGTGGATTACAAAATCCCCCGGACGGCTGCCC
Site 1 BH-sgRNA H14-B1-P6 R	AAACGGGCAGCCGTCCGGGGGATTTTGTAATCCACCGGACGG
Site 1 BH-sgRNA H14-B1-P7 F	GCACCCGTCCGTGGGATTACAAAATCCCCCGGACGGCTGCCC
Site 1 BH-sgRNA H14-B1-P7 R	AAACGGGCAGCCGTCCGGGGGATTTTGTAATCCCACGGACGG
Site 1 BH-sgRNA H14-B1-P8 F	GCACCCGTCCTGGGGATTACAAAATCCCCCGGACGGCTGCCC
Site 1 BH-sgRNA H14-B1-P8 R	AAACGGGCAGCCGTCCGGGGGATTTTGTAATCCCCAGGACGG
Site 1 BH-sgRNA H14-B2-P4 F	GCACCCGTCCGGGTTATTACAAAATCCCCCGGACGGCTGCCC

Site 1 BH-sgRNA H14-B2-P4 R	AAACGGGCAGCCGTCCGGGGGATTTTGTAATAACCCGGACGG
Site 1 BH-sgRNA H14-B2-P5 F	GCACCCGTCCGGTTGATTACAAAATCCCCCGGACGGCTGCCC
Site 1 BH-sgRNA H14-B2-P5 R	AAACGGGCAGCCGTCCGGGGGATTTTGTAATCAACCGGACGG
Site 1 BH-sgRNA H14-B2-P6 F	GCACCCGTCCGTTGGATTACAAAATCCCCCGGACGGCTGCCC
Site 1 BH-sgRNA H14-B2-P6 R	AAACGGGCAGCCGTCCGGGGGATTTTGTAATCCAACGGACGG
Site 1 BH-sgRNA H14-B2-P7 F	GCACCCGTCCTTGGGATTACAAAATCCCCCGGACGGCTGCCC
Site 1 BH-sgRNA H14-B2-P7 R	AAACGGGCAGCCGTCCGGGGGATTTTGTAATCCCAAGGACGG
Site 1 BH-sgRNA H14-B3-P4 F	GCACCCGTCCGGTTTATTACAAAATCCCCCGGACGGCTGCCC
Site 1 BH-sgRNA H14-B3-P4 R	AAACGGGCAGCCGTCCGGGGGATTTTGTAATAAACCGGACGG
Site 1 BH-sgRNA H14-B3-P5 F	GCACCCGTCCGTTTGATTACAAAATCCCCCGGACGGCTGCCC
Site 1 BH-sgRNA H14-B3-P5 R	AAACGGGCAGCCGTCCGGGGGATTTTGTAATCAAACGGACGG
Site 1 BH-sgRNA H14-B3-P6 F	GCACCCGTCCTTTGGATTACAAAATCCCCCGGACGGCTGCCC
Site 1 BH-sgRNA H14-B3-P6 R	AAACGGGCAGCCGTCCGGGGGATTTTGTAATCCAAAGGACGG

Site 2 BE3 sgRNA cloning

Site 2 WT-sgRNA F	GCACACAAGCTATCGCCGGATGCGATGC
Site 2 WT-sgRNA R	AAACGCATCGCATCCGGCGATAGCTTGT
Site 2 Hp-sgRNA H2 F	GCACGCACAAGCTATCGCCGGATGCGATGC
Site 2 Hp-sgRNA H2R	AAACGCATCGCATCCGGCGATAGCTTGTGC
Site 2 Hp-sgRNA H4 F	GCACTAGCACAAGCTATCGCCGGATGCGATGC
Site 2 Hp-sgRNA H4 R	AAACGCATCGCATCCGGCGATAGCTTGTGCTA
Site 2 Hp-sgRNA H6 F	GCACGATAGCACAAGCTATCGCCGGATGCGATGC
Site 2 Hp-sgRNA H6 R	AAACGCATCGCATCCGGCGATAGCTTGTGCTATC
Site 2 Hp-sgRNA H8 F	GCACGCGATAGCACAAGCTATCGCCGGATGCGATGC
Site 2 Hp-sgRNA H8 R	AAACGCATCGCATCCGGCGATAGCTTGTGCTATCGC
Site 2 Hp-sgRNA H10 F	GCACCGGCGATAGCACAAGCTATCGCCGGATGCGATGC
Site 2 Hp-sgRNA H10 R	AAACGCATCGCATCCGGCGATAGCTTGTGCTATCGCCG
Site 2 Hp-sgRNA H12 F	GCACTCCGGCGATAGCACAAGCTATCGCCGGATGCGATGC
Site 2 Hp-sgRNA H12 R	AAACGCATCGCATCCGGCGATAGCTTGTGCTATCGCCGGA
Site 2 Hp-sgRNA H14 F	GCACCATCCGGCGATAGCACAAGCTATCGCCGGATGCGATGC
Site 2 Hp-sgRNA H14 R	AAACGCATCGCATCCGGCGATAGCTTGTGCTATCGCCGGATG
Site 2 BH-sgRNA H12-B1-P6 F	GCACTCCGGCAATAGCACAAGCTATCGCCGGATGCGATGC
Site 2 BH-sgRNA H12-B1-P6 R	AAACGCATCGCATCCGGCGATAGCTTGTGCTATTGCCGGA
Site 2 BH-sgRNA H12-B2-P5 F	GCACTCCGGCAGTAGCACAAGCTATCGCCGGATGCGATGC
Site 2 BH-sgRNA H12-B2-P5 R	AAACGCATCGCATCCGGCGATAGCTTGTGCTACTGCCGGA
Site 2 BH-sgRNA H12-B3-P5 F	GCACTCCGGTAGTAGCACAAGCTATCGCCGGATGCGATGC
Site 2 BH-sgRNA H12-B3-P5 R	AAACGCATCGCATCCGGCGATAGCTTGTGCTACTACCGGA

Site 3 BE3 sgRNA cloning

Site 3 WT-sgRNA F	GCACACAAGGAAGTCCATCCGGCACGAG
Site 3 WT-sgRNA R	AAACCTCGTGCCGGATGGACTTCCTTGT
Site 3 Hp-sgRNA H2 F	GCACCCACAAGGAAGTCCATCCGGCACGAG
Site 3 Hp-sgRNA H2 R	AAACCTCGTGCCGGATGGACTTCCTTGTGG

Site 3 Hp-sgRNA H4 F	GCACTTCCACAAGGAAGTCCATCCGGCACGAG
Site 3 Hp-sgRNA H4 R	AAACCTCGTGCCGGATGGACTTCCTTGTGGAA
Site 3 Hp-sgRNA H6 F	GCACACTTCCACAAGGAAGTCCATCCGGCACGAG
Site 3 Hp-sgRNA H6 R	AAACCTCGTGCCGGATGGACTTCCTTGTGGAAGT
Site 3 Hp-sgRNA H8 F	GCACATGGACTTCCACAAGGAAGTCCATCCGGCACGAG
Site 3 Hp-sgRNA H8 R	AAACCTCGTGCCGGATGGACTTCCTTGTGGAAGTCCAT
Site 3 Hp-sgRNA H10 F	GCACATGGACTTCCACAAGGAAGTCCATCCGGCACGAG
Site 3 Hp-sgRNA H10 R	AAACCTCGTGCCGGATGGACTTCCTTGTGGAAGTCCAT
Site 3 Hp-sgRNA H12 F	GCACGGATGGACTTCCACAAGGAAGTCCATCCGGCACGAG
Site 3 Hp-sgRNA H12 R	AAACCTCGTGCCGGATGGACTTCCTTGTGGAAGTCCATCC
Site 3 Hp-sgRNA H14 F	GCACCCGGATGGACTTCCACAAGGAAGTCCATCCGGCACGAG
Site 3 Hp-sgRNA H14 R	AAACCTCGTGCCGGATGGACTTCCTTGTGGAAGTCCATCCGG
Site 3 BH-sgRNA H12-B1-P6 F	GCACGGATGGGCTTCCACAAGGAAGTCCATCCGGCACGAG
Site 3 BH-sgRNA H12-B1-P6 R	AAACCTCGTGCCGGATGGACTTCCTTGTGGAAGCCCATCC
Site 3 BH-sgRNA H12-B2-P5 F	GCACGGATGGGTTTCCACAAGGAAGTCCATCCGGCACGAG
Site 3 BH-sgRNA H12-B2-P5 R	AAACCTCGTGCCGGATGGACTTCCTTGTGGAAACCCATCC
Site 3 BH-sgRNA H12-B3-P5 F	GCACGGATGAGTTTCCACAAGGAAGTCCATCCGGCACGAG
Site 3 BH-sgRNA H12-B3-P5 R	AAACCTCGTGCCGGATGGACTTCCTTGTGGAAACTCATCC

Site 4 BE3 sgRNA cloning

Site 4 WT-sgRNA F	GCACCGTAACGCCCGATGCGACGC
Site 4 WT-sgRNA R	AAACGCGTCGCATCGGGCGTTACG
Site 4 BH-sgRNA H12-B3-P5 F	GCACTCGGGTACTACGACAACGTAACGCCCGATGCGACGC
Site 4 BH-sgRNA H12-B3-P5 R	AAACGCGTCGCATCGGGCGTTACGTTGTCGTAGTACCCGA

Site 5 BE3 sgRNA cloning

Site 5 WT-sgRNA F	GCACTGAACACCTTATCCGACCTA
Site 5 WT-sgRNA R	AAACTAGGTCGGATAAGGTGTTCA
Site 5 BH-sgRNA H12-B3-P5 F	GCACATAAGACATTCAACAATGAACACCTTATCCGACCTA
Site 5 BH-sgRNA H12-B3-P5 R	AAACTAGGTCGGATAAGGTGTTCATTGTTGAATGTCTTAT

Site 6 BE3 WT-sgRNA cloning (single mismatch)

OT1-1 F	GCACTTTCTCTCTCCTATCACTTC
OT1-1 R	AAACGAAGTGATAGGAGAGAGAAA
OT1-2 F	GCACAATCTCTCTCCTATCACTTC
OT1-2 R	AAACGAAGTGATAGGAGAGAGATT
OT1-3 F	GCACATACTCTCTCCTATCACTTC
OT1-3 R	AAACGAAGTGATAGGAGAGAGTAT
OT1-4 F	GCACATTGTCTCTCCTATCACTTC
OT1-4 R	AAACGAAGTGATAGGAGAGACAAT
OT1-5 F	GCACATTCACTCTCCTATCACTTC
OT1-5 R	AAACGAAGTGATAGGAGAGTGAAT
OT1-6 F	GCACATTCTGTCTCCTATCACTTC

OT1-6 R	AAACGAAGTGATAGGAGACAGAAT
OT1-7 F	GCACATTCTCACTCCTATCACTTC
OT1-7 R	AAACGAAGTGATAGGAGTGAGAAT
OT1-8 F	GCACATTCTCTGTCCTATCACTTC
OT1-8 R	AAACGAAGTGATAGGACAGAGAAT
OT1-9 F	GCACATTCTCTCACCTATCACTTC
OT1-9 R	AAACGAAGTGATAGGTGAGAGAAT
OT1-10 F	GCACATTCTCTCTGCTATCACTTC
OT1-10 R	AAACGAAGTGATAGCAGAGAGAAT
OT1-11 F	GCACATTCTCTCTCGTATCACTTC
OT1-11 R	AAACGAAGTGATACGAGAGAGAAT
OT1-12 F	GCACATTCTCTCTCCAATCACTTC
OT1-12 R	AAACGAAGTGATTGGAGAGAGAAT
OT1-13 F	GCACATTCTCTCTCCTTTCACTTC
OT1-13 R	AAACGAAGTGAAAGGAGAGAGAAT
OT1-14 F	GCACATTCTCTCTCCTAACACTTC
OT1-14 R	AAACGAAGTGTTAGGAGAGAGAAT
OT1-15 F	GCACATTCTCTCTCCTATGACTTC
OT1-15 R	AAACGAAGTCATAGGAGAGAGAAT
OT1-16 F	GCACATTCTCTCTCCTATCTCTTC
OT1-16 R	AAACGAAGAGATAGGAGAGAGAAT
OT1-17 F	GCACATTCTCTCTCCTATCAGTTC
OT1-17 R	AAACGAACTGATAGGAGAGAGAAT
OT1-18 F	GCACATTCTCTCTCCTATCACATC
OT1-18 R	AAACGATGTGATAGGAGAGAGAAT
OT1-19 F	GCACATTCTCTCTCCTATCACTAC
OT1-19 R	AAACGTAGTGATAGGAGAGAGAAT
OT1-20 F	GCACATTCTCTCTCCTATCACTTG
OT1-20 R	AAACCAAGTGATAGGAGAGAGAAT

Site 6 BE3 BH-sg RNA cloning (single mismatch)

BHOT1-1 F	GCACAGGAGGAGGAAAACAATTTCTCTCTCCTATCACTTC
BHOT1-1 R	AAACGAAGTGATAGGAGAGAGAAATTGTTTTCCTCCTCCT
BHOT1-2 F	GCACAGGAGGAGGATTACAAAATCTCTCTCCTATCACTTC
BHOT1-2 R	AAACGAAGTGATAGGAGAGAGATTTTGTAATCCTCCTCCT
BHOT1-3 F	GCACAGGAGGAGGTATACAAATACTCTCTCCTATCACTTC
BHOT1-3 R	AAACGAAGTGATAGGAGAGAGTATTTGTATACCTCCTCCT
BHOT1-4 F	GCACAGGAGGAGCAATACAAATTGTCTCTCCTATCACTTC
BHOT1-4 R	AAACGAAGTGATAGGAGAGACAATTTGTATTGCTCCTCCT
BHOT1-5 F	GCACAGGAGGACGAATACAAATTCACTCTCCTATCACTTC
BHOT1-5 R	AAACGAAGTGATAGGAGAGTGAATTTGTATTCGTCCTCCT
BHOT1-6 F	GCACAGGAGGTGGAATACAAATTCTGTCTCCTATCACTTC
BHOT1-6 R	AAACGAAGTGATAGGAGACAGAATTTGTATTCCACCTCCT

BHOT1-7 F	GCACAGGAGCAGGAATACAAATTCTCACTCCTATCACTTC
BHOT1-7 R	AAACGAAGTGATAGGAGTGAGAATTTGTATTCCTGCTCCT
BHOT1-8 F	GCACAGGACGAGGAATACAAATTCTCTGTCCTATCACTTC
BHOT1-8 R	AAACGAAGTGATAGGACAGAGAATTTGTATTCCTCGTCCT
BHOT1-9 F	GCACAGGTGGAGGAATACAAATTCTCTCACCTATCACTTC
BHOT1-9 R	AAACGAAGTGATAGGTGAGAGAATTTGTATTCCTCCACCT
BHOT1-10 F	GCACAGCAGGAGGAATACAAATTCTCTCTGCTATCACTTC
BHOT1-10 R	AAACGAAGTGATAGCAGAGAGAATTTGTATTCCTCCTGCT
BHOT1-11 F	GCACACGAGGAGGAATACAAATTCTCTCTCGTATCACTTC
BHOT1-11 R	AAACGAAGTGATACGAGAGAGAATTTGTATTCCTCCTCGT
BHOT1-12 F	GCACTGGAGGAGGAATACAAATTCTCTCTCCAATCACTTC
BHOT1-12 R	AAACGAAGTGATTGGAGAGAGAATTTGTATTCCTCCTCCA
BHOT1-13 F	GCACAGGAGGAGGAATACAAATTCTCTCTCCTTTCACTTC
BHOT1-13 R	AAACGAAGTGAAAGGAGAGAGAATTTGTATTCCTCCTCCT
BHOT1-14 F	GCACAGGAGGAGGAATACAAATTCTCTCTCCTAACACTTC
BHOT1-14 R	AAACGAAGTGTTAGGAGAGAGAATTTGTATTCCTCCTCCT
BHOT1-15 F	GCACAGGAGGAGGAATACAAATTCTCTCTCCTATGACTTC
BHOT1-15 R	AAACGAAGTCATAGGAGAGAGAATTTGTATTCCTCCTCCT
BHOT1-16 F	GCACAGGAGGAGGAATACAAATTCTCTCTCCTATCTCTTC
BHOT1-16 R	AAACGAAGAGATAGGAGAGAGAATTTGTATTCCTCCTCCT
BHOT1-17 F	GCACAGGAGGAGGAATACAAATTCTCTCTCCTATCAGTTC
BHOT1-17 R	AAACGAACTGATAGGAGAGAGAATTTGTATTCCTCCTCCT
BHOT1-18 F	GCACAGGAGGAGGAATACAAATTCTCTCTCCTATCACATC
BHOT1-18 R	AAACGATGTGATAGGAGAGAGAATTTGTATTCCTCCTCCT
BHOT1-19 F	GCACAGGAGGAGGAATACAAATTCTCTCTCCTATCACTAC
BHOT1-19 R	AAACGTAGTGATAGGAGAGAGAATTTGTATTCCTCCTCCT
BHOT1-20 F	GCACAGGAGGAGGAATACAAATTCTCTCTCCTATCACTTG
BHOT1-20 R	AAACCAAGTGATAGGAGAGAGAATTTGTATTCCTCCTCCT

Site 6 BE3 WT-sgRNA cloning (double mismatches)

OT2-1 F	GCACTATCTCTCTCCTATCACTTC
OT2-1 R	AAACGAAGTGATAGGAGAGAGATA
OT2-3 F	GCACATAGTCTCTCCTATCACTTC
OT2-3 R	AAACGAAGTGATAGGAGAGACTAT
OT2-5 F	GCACATTCAGTCTCCTATCACTTC
OT2-5 R	AAACGAAGTGATAGGAGACTGAAT
OT2-7 F	GCACATTCTCAGTCCTATCACTTC
OT2-7 R	AAACGAAGTGATAGGACTGAGAAT
OT2-9 F	GCACATTCTCTCAGCTATCACTTC
OT2-9 R	AAACGAAGTGATAGCTGAGAGAAT
OT2-11 F	GCACATTCTCTCTCGAATCACTTC
OT2-11 R	AAACGAAGTGATTCGAGAGAGAAT
OT2-13 F	GCACATTCTCTCTCCTTACACTTC

OT2-13 R	AAACGAAGTGTAAGGAGAGAGAAT
OT2-15 F	GCACATTCTCTCTCCTATGTCTTC
OT2-15 R	AAACGAAGACATAGGAGAGAGAAT
OT2-17 F	GCACATTCTCTCTCCTATCAGATC
OT2-17 R	AAACGATCTGATAGGAGAGAGAAT
OT2-19 F	GCACATTCTCTCTCCTATCACTAG
OT2-19 R	AAACCTAGTGATAGGAGAGAGAAT

Site 6 BE3 BH-sgRNA cloning (double mismatches)

BHOT2-1 F	GCACAGGAGGAGGATAACAATATCTCTCTCCTATCACTTC
BHOT2-1 R	AAACGAAGTGATAGGAGAGAGATATTGTTATCCTCCTCCT
BHOT2-3 F	GCACAGGAGGAGCTATACAAATAGTCTCTCCTATCACTTC
BHOT2-3 R	AAACGAAGTGATAGGAGAGACTATTTGTATAGCTCCTCCT
BHOT2-5 F	GCACAGGAGGTCGAATACAAATTCAGTCTCCTATCACTTC
BHOT2-5 R	AAACGAAGTGATAGGAGACTGAATTTGTATTCGACCTCCT
BHOT2-7 F	GCACAGGACCAGGAATACAAATTCTCAGTCCTATCACTTC
BHOT2-7 R	AAACGAAGTGATAGGACTGAGAATTTGTATTCCTGGTCCT
BHOT2-9 F	GCACAGCTGGAGGAATACAAATTCTCTCAGCTATCACTTC
BHOT2-9 R	AAACGAAGTGATAGCTGAGAGAATTTGTATTCCTCCAGCT
BHOT2-11 F	GCACTCGAGGAGGAATACAAATTCTCTCTCGAATCACTTC
BHOT2-11 R	AAACGAAGTGATTCGAGAGAGAATTTGTATTCCTCCTCGA
BHOT2-13 F	GCACAGGAGGAGGAATACAAATTCTCTCTCCTTACACTTC
BHOT2-13 R	AAACGAAGTGTAAGGAGAGAGAATTTGTATTCCTCCTCCT
BHOT2-15 F	GCACAGGAGGAGGAATACAAATTCTCTCTCCTATGTCTTC
BHOT2-15 R	AAACGAAGACATAGGAGAGAGAATTTGTATTCCTCCTCCT
BHOT2-17 F	GCACAGGAGGAGGAATACAAATTCTCTCTCCTATCAGATC
BHOT2-17 R	AAACGATCTGATAGGAGAGAGAATTTGTATTCCTCCTCCT
BHOT2-19 F	GCACAGGAGGAGGAATACAAATTCTCTCTCCTATCACTAG
BHOT2-19 R	AAACCTAGTGATAGGAGAGAGAATTTGTATTCCTCCTCCT

Site 6 BE3 WT-sgRNA cloning (separate double mismatches)

OT2-1S F	GCACTTTCTCTCTCCTATCACTTG
OT2-1S R	AAACCAAGTGATAGGAGAGAGAAA
OT2-2S F	GCACAATCTCTCTCCTATCACTAC
OT2-2S R	AAACGTAGTGATAGGAGAGAGATT
OT2-3S F	GCACATACTCTCTCCTATCACATC
OT2-3S R	AAACGATGTGATAGGAGAGAGTAT
OT2-4S F	GCACATTGTCTCTCCTATCAGTTC
OT2-4S R	AAACGAACTGATAGGAGAGACAAT
OT2-5S F	GCACATTCACTCTCCTATCTCTTC
OT2-5S R	AAACGAAGAGATAGGAGAGTGAAT
OT2-6S F	GCACATTCTGTCTCCTATGACTTC
OT2-6S R	AAACGAAGTCATAGGAGACAGAAT

OT2-7S F	GCACATTCTCACTCCTAACACTTC
OT2-7S R	AAACGAAGTGTTAGGAGTGAGAAT
OT2-8S F	GCACATTCTCTGTCCTTTCACTTC
OT2-8S R	AAACGAAGTGAAAGGACAGAGAAT
OT2-9S F	GCACATTCTCTCACCAATCACTTC
OT2-9S R	AAACGAAGTGATTGGTGAGAGAAT
OT2-10S F	GCACATTCTCTCTGGTATCACTTC
OT2-10S R	AAACGAAGTGATACCAGAGAGAAT

Site 6 BE3 BH-sgRNA cloning (separate double mismatches)

BHOT2-1S F	GCACAGGAGGAGGAAAACAATTTCTCTCTCCTATCACTTG
BHOT2-1S R	AAACCAAGTGATAGGAGAGAGAAATTGTTTTCCTCCTCCT
BHOT2-2S F	GCACAGGAGGAGGATTACAAAATCTCTCTCCTATCACTAC
BHOT2-2S R	AAACGTAGTGATAGGAGAGAGATTTTGTAATCCTCCTCCT
BHOT2-3S F	GCACAGGAGGAGGTATACAAATACTCTCTCCTATCACATC
BHOT2-3S R	AAACGATGTGATAGGAGAGAGTATTTGTATACCTCCTCCT
BHOT2-4S F	GCACAGGAGGAGCAATACAAATTGTCTCTCCTATCAGTTC
BHOT2-4S R	AAACGAACTGATAGGAGAGACAATTTGTATTGCTCCTCCT
BHOT2-5S F	GCACAGGAGGACGAATACAAATTCACTCTCCTATCTCTTC
BHOT2-5S R	AAACGAAGAGATAGGAGAGTGAATTTGTATTCGTCCTCCT
BHOT2-6S F	GCACAGGAGGTGGAATACAAATTCTGTCTCCTATGACTTC
BHOT2-6S R	AAACGAAGTCATAGGAGACAGAATTTGTATTCCACCTCCT
BHOT2-7S F	GCACAGGAGCAGGAATACAAATTCTCACTCCTAACACTTC
BHOT2-7S R	AAACGAAGTGTTAGGAGTGAGAATTTGTATTCCTGCTCCT
BHOT2-8S F	GCACAGGACGAGGAATACAAATTCTCTGTCCTTTCACTTC
BHOT2-8S R	AAACGAAGTGAAAGGACAGAGAATTTGTATTCCTCGTCCT
BHOT2-9S F	GCACTGGTGGAGGAATACAAATTCTCTCACCAATCACTTC
BHOT2-9S R	AAACGAAGTGATTGGTGAGAGAATTTGTATTCCTCCACCA
BHOT2-10S F	GCACACCAGGAGGAATACAAATTCTCTCTGGTATCACTTC
BHOT2-10S R	AAACGAAGTGATACCAGAGAGAATTTGTATTCCTCCTGGT

Site 6 BE3 WT-sgRNA cloning (triple mismatches)

OT3-1 F	GCACTAACTCTCTCCTATCACTTC
OT3-1 R	AAACGAAGTGATAGGAGAGAGTTA
OT3-4 F	GCACATTGAGTCTCCTATCACTTC
OT3-4 R	AAACGAAGTGATAGGAGACTCAAT
OT3-7 F	GCACATTCTCAGACCTATCACTTC
OT3-7 R	AAACGAAGTGATAGGTCTGAGAAT
OT3-10 F	GCACATTCTCTCTGGAATCACTTC
OT3-10 R	AAACGAAGTGATTCCAGAGAGAAT
OT3-13 F	GCACATTCTCTCTCCTTAGACTTC
OT3-13 R	AAACGAAGTCTAAGGAGAGAGAAT
OT3-16 F	GCACATTCTCTCTCCTATCTGATC

OT3-16 R	AAACGATCAGATAGGAGAGAGAAT
OT3-18 F	GCACATTCTCTCTCCTATCACAAG
OT3-18 R	AAACCTTGTGATAGGAGAGAGAAT

Site 6 BE3 BH-sgRNA cloning (triple mismatches)

BHOT3-1 F	GCACAGGAGGAGGTTAACAATAACTCTCTCCTATCACTTC
BHOT3-1 R	AAACGAAGTGATAGGAGAGAGTTATTGTTAACCTCCTCCT
BHOT3-4 F	GCACAGGAGGTCCAATACAAATTGAGTCTCCTATCACTTC
BHOT3-4 R	AAACGAAGTGATAGGAGACTCAATTTGTATTGGACCTCCT
BHOT3-7 F	GCACAGGTCCAGGAATACAAATTCTCAGACCTATCACTTC
BHOT3-7 R	AAACGAAGTGATAGGTCTGAGAATTTGTATTCCTGGACCT
BHOT3-10 F	GCACTCCAGGAGGAATACAAATTCTCTCTGGAATCACTTC
BHOT3-10 R	AAACGAAGTGATTCCAGAGAGAATTTGTATTCCTCCTGGA
BHOT3-13 F	GCACAGGAGGAGGAATACAAATTCTCTCTCCTTAGACTTC
BHOT3-13 R	AAACGAAGTCTAAGGAGAGAGAATTTGTATTCCTCCTCCT
BHOT3-16 F	GCACAGGAGGAGGAATACAAATTCTCTCTCCTATCTGATC
BHOT3-16 R	AAACGATCAGATAGGAGAGAGAATTTGTATTCCTCCTCCT
BHOT3-18 F	GCACAGGAGGAGGAATACAAATTCTCTCTCCTATCACAAG
BHOT3-18 R	AAACCTTGTGATAGGAGAGAGAATTTGTATTCCTCCTCCT

Site 6 BE3 WT-sgRNA cloning (separate triple mismatches)

OT3-1S F	GCACTTTCTCTCTGCTATCACTTG
OT3-1S R	AAACCAAGTGATAGCAGAGAGAAA
OT3-2S F	GCACAATCTCTCTGCTATCACTAC
OT3-2S R	AAACGTAGTGATAGCAGAGAGATT
OT3-3S F	GCACATACTCTCTGCTATCACATC
OT3-3S R	AAACGATGTGATAGCAGAGAGTAT
OT3-4S F	GCACATTGTCTCTGCTATCAGTTC
OT3-4S R	AAACGAACTGATAGCAGAGACAAT
OT3-5S F	GCACATTCACTCTGCTATCTCTTC
OT3-5S R	AAACGAAGAGATAGCAGAGTGAAT
OT3-6S F	GCACATTCTGTCTGCTATGACTTC
OT3-6S R	AAACGAAGTCATAGCAGACAGAAT
OT3-7S F	GCACATTCTCACTGCTAACACTTC
OT3-7S R	AAACGAAGTGTTAGCAGTGAGAAT
OT3-8S F	GCACATTCTCTGTGCTTTCACTTC
OT3-8S R	AAACGAAGTGAAAGCACAGAGAAT
OT3-9S F	GCACATTCTCTCAGCAATCACTTC
OT3-9S R	AAACGAAGTGATTGCTGAGAGAAT

Site 6 BE3 BH-sgRNA cloning (separate triple mismatches)

BHOT3-1S F	GCACAGCAGGAGGAAAACAATTTCTCTCTGCTATCACTTG
BHOT3-1S R	AAACCAAGTGATAGCAGAGAGAAATTGTTTTCCTCCTGCT

BHOT3-2S F	GCACAGCAGGAGGATTACAAAATCTCTCTGCTATCACTAC
BHOT3-2S R	AAACGTAGTGATAGCAGAGAGATTTTGTAATCCTCCTGCT
BHOT3-3S F	GCACAGCAGGAGGTATACAAATACTCTCTGCTATCACATC
BHOT3-3S R	AAACGATGTGATAGCAGAGAGTATTTGTATACCTCCTGCT
BHOT3-4S F	GCACAGCAGGAGCAATACAAATTGTCTCTGCTATCAGTTC
BHOT3-4S R	AAACGAACTGATAGCAGAGACAATTTGTATTGCTCCTGCT
BHOT3-5S F	GCACAGCAGGACGAATACAAATTCACTCTGCTATCTCTTC
BHOT3-5S R	AAACGAAGAGATAGCAGAGTGAATTTGTATTCGTCCTGCT
BHOT3-6S F	GCACAGCAGGTGGAATACAAATTCTGTCTGCTATGACTTC
BHOT3-6S R	AAACGAAGTCATAGCAGACAGAATTTGTATTCCACCTGCT
BHOT3-7S F	GCACAGCAGCAGGAATACAAATTCTCACTGCTAACACTTC
BHOT3-7S R	AAACGAAGTGTTAGCAGTGAGAATTTGTATTCCTGCTGCT
BHOT3-8S F	GCACAGCACGAGGAATACAAATTCTCTGTGCTTTCACTTC
BHOT3-8S R	AAACGAAGTGAAAGCACAGAGAATTTGTATTCCTCGTGCT
BHOT3-9S F	GCACTGCTGGAGGAATACAAATTCTCTCAGCAATCACTTC
BHOT3-9S R	AAACGAAGTGATTGCTGAGAGAATTTGTATTCCTCCAGCA

Deep sequencing PCR primer

Site 1 on seq F	ggagtgagtacggtgtgcACACATTTTGCCAGCCGCCA
Site 1 on seq R	gagttggatgctggatggGACGCCTTCGACCTGATGCA
Site 2 on seq F	ggagtgagtacggtgtgcTCGATGATACCGTGCGACCT
Site 2 on seq R	gagttggatgctggatggAACGGTGATCTTGCCGGATG
Site 2 ot1 seq F	ggagtgagtacggtgtgcAACTCTTCTGCCGCGCCAAT
Site 2 ot1 seq R	gagttggatgctggatggCCGTACTGAACGGTCCCCTC
Site 2 ot2 seq F	ggagtgagtacggtgtgcCCACGATGCTGATGCGCAGA
Site 2 ot2 seq R	gagttggatgctggatggACCGGCATAAGGATTTGGGC
Site 2 ot3 seq F	ggagtgagtacggtgtgcGGCCGGATGCGGCGTAAACG
Site 2 ot3 seq R	gagttggatgctggatggATGAGTTGGAATGAAATTGT
Site 2 ot4 seq F	ggagtgagtacggtgtgcTCCGCTCACGGAACTTAACC
Site 2 ot4 seq R	gagttggatgctggatggGTCGGATGCGGCATAAACGC
Site 2 ot5 seq F	ggagtgagtacggtgtgcGGGATGAACGTGCGGCATAA
Site 2 ot5 seq R	gagttggatgctggatggCGGGTTTGCGATTTATCAGG
Site 2 ot6 seq F	ggagtgagtacggtgtgcTGGTGGTGTCGAACGTAGGT
Site 2 ot6 seq R	gagttggatgctggatggAACGTGCCATTAATGTCGGA
Site 2 ot7 seq F	ggagtgagtacggtgtgcACGCCTTATCCGGCCTACAA
Site 2 ot7 seq R	gagttggatgctggatggGCAAGCCATTCGATGCAGCA
Site 2 ot8 seq F	ggagtgagtacggtgtgcGCGAATACATTGCATATCGA
Site 2 ot8 seq R	gagttggatgctggatggTGCCGGATTCGGCGTGAACG
Site 2 ot9 seq F	ggagtgagtacggtgtgcAAATCGGCAATCAGCGTTAG
Site 2 ot9 seq R	gagttggatgctggatggATGTGCGCCGGGTGTTTAAT
Site 3 on seq F	ggagtgagtacggtgtgcGGGCAACAACTTACAGTCAG
Site 3 on seq R	gagttggatgctggatggCAATTGCCCGCTATTTTCCT
Site 3 ot1 seq F	ggagtgagtacggtgtgcTGGAGAAACATCTTCGATTG

Site 3 ot1 seq R	gagttggatgctggatggCCAAACGCGGCTGCTGGATA
Site 4 on seq F	ggagtgagtacggtgtgcGCAGGCGATCATCGAGCAAA
Site 4 on seq R	gagttggatgctggatggCGCGAAAGGATCGCAGTTGG
Site 4 ot1 seq F	ggagtgagtacggtgtgcAGCGGGAAGTTTGCCTGGAA
Site 4 ot1 seq R	gagttggatgctggatggCTAACAGCTTCGGCTTCGGC
Site 4 ot2 seq F	ggagtgagtacggtgtgcCAAACGCAAAACAGGCCAGA
Site 4 ot2 seq R	gagttggatgctggatggAAGGCGGGTCGCTTTGTTGG
Site 4 ot3 seq F	ggagtgagtacggtgtgcCCGGATAGCATCTTCCGCGT
Site 4 ot3 seq R	gagttggatgctggatggGCACAACTGCCGGATTCGGC
Site 4 ot4 seq F	ggagtgagtacggtgtgcGGGAATCGGTATTCAGCGAA
Site 4 ot4 seq R	gagttggatgctggatggACAGTATTTTGTCCAGCCGT
Site 4 ot5 seq F	ggagtgagtacggtgtgcCCTTCGTGCCGGATGCGCTT
Site 4 ot5 seq R	gagttggatgctggatggCCTACACGCTGCGATTTTGT
Site 5 on seq F	ggagtgagtacggtgtgcCGTAAAGTGGTTGGGGTTTAG
Site 5 on seq R	gagttggatgctggatggGCAGGGTAAGACCAATGAGT
Site 5 ot1 seq F	ggagtgagtacggtgtgcCCTGACCGAAACGGCCAATT
Site 5 ot1 seq R	gagttggatgctggatggCTCTTATCTCGAAGAGGATG
Site 5 ot2 seq F	ggagtgagtacggtgtgcCGCGCATCAATAAAAATGGC
Site 5 ot2 seq R	gagttggatgctggatggCAACTTTCTGGCTTAAGTGG
Site 5 ot3 seq F	ggagtgagtacggtgtgcGGTAACCACAGCGTTTACGT
Site 5 ot3 seq R	gagttggatgctggatggGAAAGCCTTATGGCTGACAG
Site 5 ot4 seq F	ggagtgagtacggtgtgcCGCATCAGGCATTTATCGCC
Site 5 ot4 seq R	gagttggatgctggatggGTATATTGGGCAGGCGTAAT
Site 5 ot5 seq F	ggagtgagtacggtgtgcGTCCGTCGGTGGTGCTATGT
Site 5 ot5 seq R	gagttggatgctggatggGTAGGTCAGATAAGGCGCTC
Site 5 ot6 seq F	ggagtgagtacggtgtgcGCATTTGCAGCGCATTAAAG
Site 5 ot6 seq R	gagttggatgctggatggCGATATAGGGTGAAGCGTAT
Site 5 ot7 seq F	ggagtgagtacggtgtgcGGTTATGGCCACAAGAGTAA
Site 5 ot7 seq R	gagttggatgctggatggCTTATCCGCCCTACGCGGT
Site 5 ot8 seq F	ggagtgagtacggtgtgcCGGTTCCAGGTCGGCATATA
Site 5 ot8 seq R	gagttggatgctggatggCTGAACTCCATCTTCCGCCG
Site 5 ot9 seq F	ggagtgagtacggtgtgcCCTACGGTTCGCTATCTCTC
Site 5 ot9 seq R	gagttggatgctggatggCATAACGCCGCCAGAAATGC
Site 5 ot10 seq F	ggagtgagtacggtgtgcCTGAATTTTACTCGGGGCAG
Site 5 ot10 seq R	gagttggatgctggatggCTGACACGGTTTTCCCCTCA
Site 6 seq F	ggagtgagtacggtgtgcACACCATCGTCACAGCCTTCG
Site 6 seq R	gagttggatgctggatggTGCGTCGATCTTCCGCGGTA

Primers for cloning ABE7.10 sgRNA, Sanger sequencing, and deep sequencing PCR

Site 7 BH-sgRNA R	AAACGCATGAACAAAGCCTACGTTttgtAACACGGGCTTT
Site 7 BH-sgRNA F	GCACGACGCGCGCTGCacaaGCAATACGCGTCCAGAATGG
Site 7 WT-sgRNA F	AAACCCATTCTGGACGCGTATTGC
Site 7 WT-sgRNA R	GCACGCAATACGCGTCCAGAATGG

Site 8 BH-sgRNA F	AAACCGAAAGCGATTCCGGTACAGttgtCTGCGTCGGAAT
Site 8 BH-sgRNA R	GCACATTCCGACGCAGacaaCTGTACCGGAATCGCTTTCG
Site 8 WT-sgRNA F	AAACCGAAAGCGATTCCGGTACAG
Site 8 WT-sgRNA R	GCACCTGTACCGGAATCGCTTTCG
Site 9 BH-sgRNA R	GCACTCCGGACGCCGGacaaCCGATATCCGGATAACGCAA
Site 9 BH-sgRNA F	AAACTTGCGTTATCCGGATATCGGttgtCCGGCGTCCGGA
Site 9 WT-sgRNA F	GCACCCGATATCCGGATAACGCAA
Site 9 WT-sgRNA R	AAACTTGCGTTATCCGGATATCGG
Site 10 BH-sgRNA F	GCACCGTTGGCGATCCacaaGGACTACCAACGTTCACCAC
Site 10 BH-sgRNA R	AAACGTGGTGAACGTTGGTAGTCCttgtGGATCGCCAACG
Site 10 WT-sgRNA F	GCACGGACTACCAACGTTCACCAC
Site 10 WT-sgRNA R	AAACGTGGTGAACGTTGGTAGTCC
Site 11 BH-sgRNA F	GCACATCCAACGTAGTacaaACTGTATTGGATGGTATGAC
Site 11 BH-sgRNA R	AAACGTCATACCATCCAATACAGTttgtACTACGTTGGAT
Site 11 WT-sgRNA F	GCACACTGTATTGGATGGTATGAC
Site 11 WT-sgRNA R	AAACGTCATACCATCCAATACAGT
Site 7 on seq F	ggagtgagtacggtgtgcCGATCCCTTTTTGTCCGTCG
Site 7 on seq R	gagttggatgctggatggGGTCATTGCGGGAGGCCTG
Site 7 ot1 seq F	ggagtgagtacggtgtgcCCAATGCCTTTCTGACCATCG
Site 7 ot1 seq R	gagttggatgctggatggGGTGTTTGCTGGCGGTCTG
Site 7 ot2 seq F	ggagtgagtacggtgtgcCACCAGATAACGGAGATCGGG
Site 7 ot2 seq R	gagttggatgctggatggCCCATGCTGGGTAAATATAAAGCC
Site 8 on seq F	ggagtgagtacggtgtgcCGGATTCGCTGGAGACGATG
Site 8 on seq R	gagttggatgctggatggCCAGTGTCGGCGATATTGTCAT
Site 8 ot1 seq F	ggagtgagtacggtgtgcCACTAACAATGGCGGTGTTTTCG
Site 8 ot1 seq R	gagttggatgctggatggCCAGAAGCCAAGCTGAATACCC
Site 8 ot2 seq F	ggagtgagtacggtgtgcGCTTCTTGCTGACGAGGAGT
Site 8 ot2 seq R	gagttggatgctggatggCTTAAATCCAGCATCCTGGCAG
Site 7 on Sanger seq F	GCTTCGCGGAGGTATCAGAG
Site 7 on Sanger seq R	CTGGGAACGTGGTTCTTCGG
Site 8 on Sanger seq F	CGGGTAGATGGTTATCGCCGAC
Site 8 on Sanger seq R	GATGCTGCAGGGCAAACTCC
Site 9 on Sanger seq F	GCCGGTGTAGACATCACCTT
Site 9 on Sanger seq R	CCCTCGTGGTTATTCTGTTTGC
Site 10 on Sanger seq F	GTGACAGTTCGCGCATCCAC
Site 10 on Sanger seq R	CGACAAGCAGCGGATGGTGT
Site 11 on Sanger seq F	GTTGTCGCCGTTGTGTTGCC
Site 11 on Sanger seq R	CGACGCCCTACTTTCGCAGG

Primers for cloning BE3 sgRNA and deep sequencing PCR of HEK293T

pV2-CWT F	GACCCCCTCCACCCCGCCTCgttttagagctagaaatagcaagttaaaat
pV2-CWT R	GAGGCGGGGTGGAGGGGGTCggtgtttcgtcctttccacaag
pV2-CBH F	AGGTCacaaGACCCCCTCCACCCCGCCTCgttttagagctagaaatagcaagttaaaat

pV2-CBH R	AGGGGGTCttgtGACCTTTTCCACggtgtttcgtcctttccacaag
pH4-CWT F	GGCACTGCGGCTGGAGGTGGgttttagagctagaaatagcaagttaaaat
pH4-CWT R	CCACCTCCAGCCGCAGTGCCggtgtttcgtcctttccacaag
pH4-CBH F	ATGCCacaaGGCACTGCGGCTGGAGGTGGgttttagagctagaaatagcaagttaaaat
pH4-CBH R	GCAGTGCCttgtGGCATCACGGCTggtgtttcgtcctttccacaag
VEGFA ON F	ggagtgagtacggtgtgcTCGGCTCGGCTTCCCCCG
VEGFA ON R	gagttggatgctggatggCAGCCCCAGCTACCACCTCC
VEGFA OFF1 F	ggagtgagtacggtgtgcGCCCAGATCCTACAAGTAACAG
VEGFA OFF1 R	gagttggatgctggatggCGAGTTCCTGGCAATGCTAA
VEGFA OFF2 F	ggagtgagtacggtgtgcTTGTCCAGGAACCCCTAGCC
VEGFA OFF2 R	gagttggatgctggatggCCTACTTCTAATCCATCCTTCCTTC
VEGFA OFF3 F	ggagtgagtacggtgtgcCTTCCATACCAGCAGCAGTT
VEGFA OFF3 R	gagttggatgctggatggGGCACTCACCTCAGCTCCT
VEGFA OFF4 F	ggagtgagtacggtgtgcCTGATTCTACACCATGGTGAGT
VEGFA OFF4 R	gagttggatgctggatggTAAGCCAGGAGTTCCCAACC
HEK ON F	ggagtgagtacggtgtgcGCAGAGGGTCCAAAGCAGGA
HEK ON R	gagttggatgctggatggGGGCTCCTTTCAACCCGAAC
HEK OFF1 F	ggagtgagtacggtgtgcCTGGGGCTGAAGATCCCTAG
HEK OFF1 R	gagttggatgctggatggCCTCCTCGGAGTCCTCAAGT
HEK OFF2 F	ggagtgagtacggtgtgcGAGGCATTGGGCAGGGGAAG
HEK OFF2 R	gagttggatgctggatggGTGGTGCAGTGCACTGAAGAG
HEK OFF3 F	ggagtgagtacggtgtgcGGCGGGAAAAGAGAAAAGCC
HEK OFF3 R	gagttggatgctggatggTGGCATTGTCCCAGCTAAGC
HEK OFF4 F	ggagtgagtacggtgtgcTGGAATCACCTGCACCCGGA
HEK OFF4 R	gagttggatgctggatggGAAATACGGGCTTAGGTGTGGG
HEK OFF6 F	ggagtgagtacggtgtgcGATGGCTTATGTCACCATTCCC
HEK OFF6 R	gagttggatgctggatggGGTGACATGTTTTACCCACATCC
HEK OFF7 F	ggagtgagtacggtgtgcGCAGGCAGTGGTCCGCAA
HEK OFF7 R	gagttggatgctggatggCTGGTAGACCACCTGGCCGG
HEK OFF9 F	ggagtgagtacggtgtgcGGGGGAGAAAAAGGCCATTC
HEK OFF9 R	gagttggatgctggatggGTAGAGAGTCTTGTTTCCTGTAGAG
HEK OFF10 F	ggagtgagtacggtgtgcGAGAAGGTAGTAGGAATCCCATTG
HEK OFF10 R	gagttggatgctggatggCCTGCAGAACATCAACTTTTGTG

Note: The bridging and specific site sequences for deep sequencing are showed in lowercase and capital letters, respectively.
