# Supplementary material for: Improving the Precision of Base Editing by Bubble Hairpin Single Guide RNA
Source: mBio. 2021 Apr 20;12(2):e00342-21. doi: 10.1128/mBio.00342-21 (PMC8092237; doi:10.1128/mBio.00342-21)
Supplement: TABLE S5 [file mBio.00342-21-st005.pdf]

**TABLE S5** Bacterial strains used in this study.

| *E. coli* strain | Description | Source/Reference |
|---|---|---|
| DH10B | Host strain for general cloning | Invitrogen |
| BL21(DE3) | Strain for base editing | Invitrogen |
| DB3.1 | Host strain for BEs plasmid construction | Invitrogen |
