# Supplementary material for: Improving the Precision of Base Editing by Bubble Hairpin Single Guide RNA
Source: mBio. 2021 Apr 20;12(2):e00342-21. doi: 10.1128/mBio.00342-21 (PMC8092237; doi:10.1128/mBio.00342-21)
Supplement: TABLE S6 [file mBio.00342-21-st006.pdf]

TABLE S6 Plasmids used in this study.

Plasmid	Description	Source
pEcBE3	Plasmid for BE3 sgRNA cloning	1
pCMV-ABE7.10	Plasmid for pEcABE7.10 construction	Addgene
pEcABE7.10	Plasmid for ABE7.10 sgRNA cloning	This work

REFERENCE

1. Zheng K, Wang Y, Li N, Jiang FF, Wu CX, Liu F, Chen HC, Liu ZF. 2018. Highly efficient base editing in bacteria using a Cas9-cytidine deaminase fusion. Commun Biol 1:32.
