# Supplementary material for: Improving the Precision of Base Editing by Bubble Hairpin Single Guide RNA
Source: mBio. 2021 Apr 20;12(2):e00342-21. doi: 10.1128/mBio.00342-21 (PMC8092237; doi:10.1128/mBio.00342-21)
Supplement: TABLE S7 [file mBio.00342-21-st007.pdf]

TABLE S7 Protospacer and PAM sequences for the on- and off-target *E. coli* genomic loci studied in this work.

Site		Sequence (5'-3')
Site 1	ON	AATCCCCCGGACGGCTGCCCAGG
Site 2	ON	GCTATCGCCGGATGCGATGCTGG
Site 2	OT1	AGCATCGCCGGATGCGATGCTGG
Site 2	OT2	GCTAGCGCCGGATGCGACGCCGG
Site 2	OT3	ATTATAGCCGGATGCGATGCTGG
Site 2	OT4	CTGGTCGCCGGATGCGATGCTGG
Site 2	OT5	ACTTTTGTCGGATGCGATGCTGG
Site 2	OT6	CGCATTGCCGGATGCGATGCTGG
Site 2	OT7	GATCGTGCCGGATGCGATGCTGG
Site 2	OT8	TTTATCGCCTGATGCGACGCTGG
Site 2	OT9	GCTGTGGCAGGATGCGTTGCAGG
Site 3	ON	GGAAGTCCATCCGGCACGAGCGG
Site 3	OT1	TGAAGTCAATCCCGCACGAGTGG
Site 4	ON	CGTAACGCCCGATGCGACGCTGG
Site 4	OT1	CGTAACGTCGGATGCGACGCTGG
Site 4	OT2	CTCAACGCCTGATGCGACGCTGG
Site 4	OT3	TTTATCGCCTGATGCGACGCTGG
Site 4	OT4	GCTAGCGCCGGATGCGACGCCGG
Site 4	OT5	TTCAACGCCTGATGCGACGCTGG
Site 5	ON	TGAACACCTTATCCGACCTACGG
Site 5	OT1	TGAACGCCTTATCCGACCTACGG
Site 5	OT2	TAAACGCCTTATCCGACCTACGG
Site 5	OT3	TAAACGCCTTATCCGACCTACGG
Site 5	OT4	TGAACGCCTTATCCGATCTACGG
Site 5	OT5	CGAGCGCCTTATCCGACCTACGG
Site 5	OT6	TGAACGCCTTATCCGACTTACAG
Site 5	OT7	TGAACGCCTTATCCGGCCTACGG
Site 5	OT8	TGAACGCCTTATCCGGCCTACGG
Site 5	OT9	CGAGCGCCTTATCCGAGCTACGG
Site 5	OT1	TGAACGCCTGATCCGGCCTACGG
Site 6	ON	ATTCTCTCTCCTATCACTTCTGG
Site 7	ON	GCAATACGCGTCCAGAATGGCGG
Site 7	OT1	GCGATACGCGTCCAGAATGGCGG
Site 7	OT2	GCAATACGTGTCCCGAGCGGTAG
Site 8	ON	CTGTACCGGAATCGCTTTCGCGG
Site 8	OT1	CTGTACCGTTATCGCTTTCGCGG
Site 8	OT2	CAGTACCGGAATCGGATTTGCAG
